# *APOE* and genetic risk variants influence Alzheimer's disease onset in carriers of an extra copy of *APP*, with and without Down syndrome

**DOI:** 10.1002/alz.71738

**Published:** 2026-08-08

**Authors:** Joan Groeneveld, Danna Perlaza, Clàudia Olivé, Lou Grangeon, Niccolo Tesi, Aude Nicolas, Chenyang Jiang, Itziar de Rojas, David Wallon, Stéphane Rousseau, Marta Rovira, Diego Real de Asúa, Fernando Moldenhauer, Ruihao Mu, Kévin Cassinari, Aline Zarea, Joaquim Aumatell Escabias, Jean‐Charles Lambert, Yolande A. L. Pijnenburg, Marc Hulsman, Everard G. B. Vijverberg, Johannes Levin, Mathias Jucker, Eric McDade, Juan Fortea, Henne Holstege, Flora H. Duits, Lisa Vermunt, Maulikkumar Patel, Matthew Johnson, Alan E. Renton, Alison M. Goate, Carlos Cruchaga, Cyril Pottier, Maria Victoria Fernandez, Olivia Belbin, Gael Nicolas, Oriol Dols‐Icardo, Sven J. van der Lee, Dominantly Inherited Alzheimer Network

**Affiliations:** ^1^ Genomics of Neurodegenerative Diseases and Aging, Human Genetics, Amsterdam UMC location VUmc Vrije Universiteit Amsterdam Amsterdam the Netherlands; ^2^ Alzheimer Center Amsterdam, Neurology, Amsterdam UMC Vrije Universiteit Amsterdam Amsterdam the Netherlands; ^3^ Neurodegeneration Amsterdam Neuroscience Amsterdam the Netherlands; ^4^ Sant Pau Memory Unit, IR SANT PAU Hospital de la Santa Creu i Sant Pau Barcelona Spain; ^5^ Network Center for Biomedical Research in Neurodegenerative Diseases (CIBERNED) Madrid Spain; ^6^ Ace Alzheimer Center Barcelona Universitat Internacional de Catalunya (UIC) Barcelona Spain; ^7^ Doctorate in Biotechnology, Facultat de Farmàcia i Ciències de l'Alimentació Universitat de Barcelona Barcelona Spain; ^8^ Inserm U1245 and, Department of Neurology and CNRMAJ Univ Rouen Normandie, Normandie Univ, CHU Rouen Rouen France; ^9^ Delft Bioinformatics Lab Delft University of Technology Delft the Netherlands; ^10^ Inserm, U1167‐RID‐AGE Facteurs de risque et déterminants moléculaires des maladies liées au vieillissement Univ. Lille, CHU Lille, Institut Pasteur de Lille Lille France; ^11^ Univ Rouen Normandie, Normandie Univ, CHU Rouen Inserm U1245 and Department of Genetics and CNRMAJ Rouen France; ^12^ Adult Down Syndrome Unit, Department of Internal Medicine Hospital Universitario de La Princesa Instituto de Investigación Sanitaria Hospital Universitario de La Princesa Madrid Spain; ^13^ Instituto de Investigación Sanitaria Hospital Universitario de La Princesa Madrid Spain; ^14^ Department of Neurology Ludwig‐Maximilians‐Universität München Munich Germany; ^15^ Site Munich German Center for Neurodegenerative Diseases Munich Germany; ^16^ Munich Cluster for Systems Neurology (SyNergy) Munich Germany; ^17^ German Center for Neurodegenerative Diseases (DZNE) Tübingen Germany; ^18^ Hertie Institute for Clinical Brain Research University of Tübingen Tübingen Germany; ^19^ Department of Neurology Washington University School of Medicine St. Louis Missouri USA; ^20^ Barcelona Down Medical Center Fundació Catalana Síndrome de Down Barcelona Spain; ^21^ VIB Center for Brain and Disease Research Leuven Belgium; ^22^ Department of Neurosciences Leuven Brain Institute KU Leuven Leuven Belgium; ^23^ Neurochemistry Laboratory Department of Laboratory Medicine, Amsterdam UMC Vrije Universiteit Amsterdam Amsterdam the Netherlands; ^24^ Department of Psychiatry Washington University School of Medicine St. Louis Missouri USA; ^25^ Neurogenomics and Informatics Center Washington University School of Medicine St. Louis Missouri USA; ^26^ Ronald M. Loeb Center for Alzheimer's Disease Icahn School of Medicine at Mount Sinai New York New York USA; ^27^ Department of Genetics and Genomic Sciences Icahn School of Medicine at Mount Sinai New York New York USA; ^28^ Nash Family Department of Neuroscience Icahn School of Medicine at Mount Sinai New York New York USA; ^29^ Hope Center for Neurological Disorders Washington University School of Medicine St. Louis Missouri USA; ^30^ Knight Alzheimer Disease Research Center Washington University School of Medicine St. Louis Missouri USA

**Keywords:** Alzheimer's disease, amyloid precursor protein duplication, apolipoprotein E, autosomal dominant Alzheimer's disease, disease onset, Down syndrome, Down syndrome‐associated Alzheimer's disease, polygenic risk score

## Abstract

**INTRODUCTION:**

An extra copy of the amyloid precursor protein (*APP*) gene causes autosomal dominant Alzheimer's disease (AD) and AD in Down syndrome (DS), but the factors underlying variability in age at onset (AAO) remain unclear. We investigated whether sporadic AD risk variants modify AAO.

**METHODS:**

We analyzed clinical and genetic data from 100 *APP* duplication (*APP*dup) carriers and 957 individuals with DS. Cox models assessed associations of apolipoprotein E (*APOE*) ε2 and ε4 and the AD genetic risk score (AD‐GRS; excluding *APOE* and chromosome 21 variants) with AAO.

**RESULTS:**

Mean AAO was earlier in *APP*dup than DS (51 ± 7 vs. 53 ± 6 years; *P =* 0.0005). *APOE* ε2 delayed onset (hazard ratio [HR] = 0.47, *P <* 0.0001), whereas *APOE* ε4 (HR = 1.5, *P =* 0.0003) and higher AD‐GRS (HR = 1.3 per standard deviation, *P <* 0.0001) accelerated onset. Predicted median AAO differed by 10 years between lowest and highest genetic risk.

**DISCUSSION:**

Sporadic AD genetic risk factors are important modifiers of AAO in *APP*dup and DS, explaining part of the marked variability in onset.

## Highlights

- Sporadic Alzheimer's disease (AD) genetic variants modify AD onset in amyloid precursor protein duplications and Down syndrome.
- Apolipoprotein E (*APOE*) ε2 delays AD onset, while *APOE* ε4 and higher AD genetic risk score accelerate onset.
- Lowest versus highest genetic risk predicts an ≈ 10‐year difference in AD onset.
- Amyloid beta, angiogenesis, and endocytosis pathways influence timing of AD onset.
- Trials in presymptomatic individuals should consider sporadic AD genetic risk.

## 1 INTRODUCTION

An extra copy of the amyloid precursor protein *(APP)* gene on chromosome 21 leads to overproduction of abnormal amyloid beta (Aβ), causing early‐onset Alzheimer's disease (AD) and/or cerebral amyloid angiopathy (CAA).^1^ The most common cause is de novo trisomy 21, responsible for Down syndrome (DS), a condition that includes intellectual disability and cardiac malformations.^2^, ^3^, ^4^ In rare cases, a small segment of chromosome 21 is duplicated without the DS critical region (small distal region on 21q22.13), leading to autosomal dominant AD (ADAD). Carriers typically present with cognitive decline, hemorrhages, or seizures.^5^, ^6^ In DS, early recognition of AD and/or CAA is complicated by intellectual disability and other co‐morbidities.^7^ In both ADAD *APP* duplications (*APP*dup) and DS‐AD, the age at onset (AAO) varies widely (40–65 years), making it difficult to predict AAO.^8^, ^9^, ^10^, ^11^, ^12^, ^13^, ^14^ This heterogeneity in AAO likely reflects a combination of environmental and genetic factors. Identifying modifiers of AAO in *APP*dup and DS is crucial for care planning and optimizing the timing of treatment initiation for emerging disease‐modifying therapies.

Among established genetic risk factors for AD, the apolipoprotein E (*APOE*) genotype is most prominent.^15^ Studies investigating *APOE* across different causes of ADAD reported that the *APOE* ε4 allele accelerated onset and the *APOE* ε2 allele delayed onset, with inconsistent significance.^12^, ^16^, ^17^, ^18^, ^19^ In *APP*dup carriers, no significant effect of *APOE* was found, nor an association of duplication length or nearby duplicated genes with AAO or specific symptoms, possibly due to small sample size.^14^ In DS, *APOE* ε4 has been linked to earlier AD onset and corresponding cerebrospinal fluid (CSF) changes, although associations with clinical onset measures have not been consistent.^20^, ^21^, ^22^ These findings suggest that the *APOE* genotype may influence the disease trajectory in individuals carrying an additional copy of *APP*.

Genome‐wide association studies (GWASs) have identified > 80 other AD risk variants beyond *APOE*.^23^ Aggregating the effects of these variants into an AD genetic risk score (AD‐GRS) provides a measure of cumulative genetic susceptibility to AD, which has been consistently associated with AAO. In ADAD and DS studies, the AD‐GRS has been associated with CSF biomarkers and cognitive decline,^22^, ^24^, ^25^ implying potential influence on AAO. These genetic variants are implicated in different biological pathways.^26^ Constructing pathway‐specific GRSs (pGRSs) allows us to determine which biological mechanisms most strongly modify AAO, thereby highlighting the pathways most relevant in individuals with an extra *APP* copy.

Although *APOE* and AD‐GRS have been examined in DS‐AD and ADAD, including *APP*dup, their contribution to variability in AAO remains unclear. Here, we hypothesized that genetic risk modifiers of AD, including *APOE* and AD‐GRS, contribute to the observed variation in AAO among individuals with an extra *APP* copy. Our aim was to investigate how these factors influence AAO in *APP*dup and DS individuals, to determine whether these effects are comparable across the two groups, and to identify specific biological pathways underlying this variability.

**RESEARCH IN CONTEXT**

1. **Systematic review**: Literature on Alzheimer's disease (AD) in amyloid precursor protein (*APP*) duplications (*APP*dup) and Down syndrome (DS) shows that the age at onset (AAO) varies between 40 and 65 years. Several studies suggest an earlier onset in apolipoprotein E (*APOE*) ε4 carriers, with no other major contributors to this variability. Genome‐wide studies in sporadic AD identified > 80 risk loci, and AD genetic risk scores (AD‐GRS) influence both disease risk and AAO. However, the role of the AD‐GRS as an AAO modifier in individuals with an extra *APP* copy has not been examined.
2. **Interpretation**: AAO was earlier in *APP*dup than DS with similar distributions. *APOE* ε2 delayed onset, while *APOE* ε4 and a higher AD‐GRS accelerated onset. Lowest versus highest genetic risk predicted an ≈10‐year AAO difference. Amyloid beta, angiogenesis, and endocytosis pathways showed the strongest AAO effects.
3. **Future directions**: *APOE* and polygenic risk may improve AAO prediction and trial design in individuals with an extra *APP* copy.

## 2 METHODS

### 2.1 Study design

In this multicenter retrospective cohort study, individuals with an additional copy of *APP* were selected from the European Alzheimer & Dementia Biobank (EADB),^23^ the Amsterdam Dementia Cohort (ADC),^27^ the Netherlands Brain Bank, the French National Reference Centre for Patients with Early‐Onset Alzheimer's Disease (CNRMAJ; Rouen University Hospital), the Dominantly Inherited Alzheimer Network Observational Study (DIAN Obs),^28^ the Ace Alzheimer Center Barcelona (ACE), Down Alzheimer's Barcelona Neuroimaging Initiative (DABNI),^29^ and the Hospital Universitario de la Princesa (HUP) in Madrid through diverse methods. All studies were approved by their local medical ethical committee.

### 2.2 Population

All individuals carrying an *APP*dup were included if clinical and genetic data were available and when the duplication did not extend across the entire DS critical region (distal 21q22.13).^30^ Previously reported *APP*dup carriers with available AAO and *APOE* genotype were additionally included after a PubMed literature search (search strategy described in the Supplementary Methods in supporting information). Individuals with DS were included if they had partial or full trisomy 21 including the triplication of *APP*, known AD status, and available genetic data.

### 2.3 Procedures

Clinical characteristics were obtained from medical records or research databases and included self‐reported sex, AD status, clinical symptoms, symptomatic AAO, age at AD diagnosis, or age at last visit. For *APP*dup carriers, AAO was defined as age at first symptom onset as indicated by the patient or patient's family. These symptoms include cognitive decline, hemorrhages, or seizures. For DS individuals, AAO corresponded to the age at (prodromal) AD diagnosis based on consensus between neurologists and neuropsychologists after independent visits. Progression to prodromal AD was defined as relevant cognitive or behavioral changes without interference in daily functioning (details in DABNI protocol).^29^ For DS individuals with established AD at first study visit, age at that visit was used as AAO.

DNA was isolated from whole blood samples or brain tissue. High‐density single nucleotide polymorphism (SNP) arrays were used for all cohorts, except for the DIAN samples, which underwent short‐read whole genome sequencing (WGS). Genotyping, imputation, sequencing, and detection of *APP*dup or trisomy 21 are described in the Supplementary Methods. When available, *APP*dup size and additional duplicated genes were recorded.

The *APOE* alleles (ε2, ε3, and ε4) and common genotypes (ε2/ε2, ε2/ε3, ε3/ε3, ε3/ε4, ε2/ε4, and ε4/ε4) were defined using two SNPs (rs429358, rs7412). The ε1 allele was not observed. For analyses, *APOE* ε2 carriers (ε2/ε2, ε2/ε3) and *APOE* ε4 carriers (ε3/ε4, ε2/ε4, ε4/ε4) were compared to the reference genotype ε3/ε3. Individuals with *APOE* ε2/ε4 were assigned to the ε4 group, consistent with previous evidence of increased AD risk compared to ε3/ε3.^31^

The AD‐GRS was calculated using genome‐wide significant variants from the most recent AD‐GWAS.^23^ Of 83 SNPs (excluding the *APOE* locus), 6 were removed from all samples, as 2 were located on chromosome 21 and 4 had low imputation quality (*R^2^ <* 0.3; Supplementary Methods). The AD‐GRS was calculated with the remaining 77 SNPs as the sum of the variant dosages weighted by the effect estimates from the AD‐GWAS. This was performed for *APP*dup carriers with available genetic data and for all DS individuals. The AD‐GRS was standardized (mean = 0, standard deviation [SD] = 1) within each population subgroup. In seven DIAN samples, one missing SNP (rs62374257) was imputed to the minor‐allele frequency from GnomAD to allow complete AD‐GRS calculation.^32^

pGRSs were constructed for the five major AD‐related pathways: Aβ metabolism, immune response, endocytosis, cholesterol/lipid metabolism, and angiogenesis.^26^ Each pGRS incorporated pathway‐specific variant weights to the original AD‐GRS calculation (Table S1 in supporting information).^26^

### 2.4 Outcomes

The primary outcome was symptomatic AAO of AD. Because AAO is more difficult to determine in individuals with DS than in those without intellectual disability,^7^ we attempted to match the definition of AAO between cohorts as follows: (1) for *APP*dup carriers, AAO corresponded to the age at first symptom onset; (2) for DABNI, AAO reflected the age at prodromal AD diagnosis or, for individuals with dementia at first visit, age at that visit; and (3) for ACE/HUP, AAO corresponded to the age at clinical AD dementia diagnosis. In the following sections, these measures are collectively referred to as AAO.

### 2.5 Statistical analysis

All analyses were performed in R version 4.4.1. Individuals unaffected at last follow‐up were censored at that age. Descriptive statistics were used to compare *APP*dup carriers and DS individuals. We used *t* tests for age and Pearson chi‐squared tests for dichotomous data. Differences in AAO were assessed using Kaplan–Meier curves and log‐rank tests, and AAO variance differences were evaluated with the Levene test.

Cox proportional hazard models assessed associations of *APOE* ε2, *APOE* ε4, and AD‐GRS with AAO. Because AD‐GRS was not available for all individuals, Model 1 included *APOE* ε2 and ε4 only, whereas Model 2 additionally included AD‐GRS. To account for different AAO definitions, cohort was included as a categorical covariate (0 = *APP*dup, 1 = DABNI, 2 = ACE/HUP). Sex and genetic principal components were not included. Hazard ratios (HRs) with 95% confidence intervals (CIs) and *p* values were reported. Potential interactions between *APOE* and AD‐GRS were tested in separate models.

Analyses were conducted in the total cohort and stratified by *APP*dup and DS groups. The DS cohorts were further stratified by DABNI and ACE/HUP to assess differences in *APOE* and AD‐GRS effects on AAO. Single‐variant analyses were performed for all AD‐GRS variants within the *APP*dup, DABNI, and ACE/HUP cohorts separately, with effects aligned to the GWAS effect allele. Cohort‐specific estimates were pooled using an inverse variance weighted fixed‐effect meta‐analysis. Genomic inflation was assessed using the genomic inflation factor calculated from the meta‐analysis *p* values, and calibration was visualized with a QQ plot. Effect‐size concordance was assessed using beta–beta plots.

Predicted median AAO with 95% CIs were calculated, and cumulative incidence curves were constructed using the riskRegression package.^33^ These curves were generated for *APP*dup and DS separately and were based on: (1) *APOE* genotypes (ε2, ε3/ε3, and ε4), (2) AD‐GRS values at five levels (lowest: –2 SD from the mean, low: –1 SD, average: 0 SD, high: +1 SD, and highest: +2 SD), and (3) two extreme risk scenarios combining *APOE* and AD‐GRS (lowest risk: *APOE* ε2 and lowest AD‐GRS; highest risk: *APOE* ε4 and highest AD‐GRS). Additionally, AD‐GRS effects within each *APOE* genotype were illustrated by computing similar cumulative incidence curves.

To assess pathway‐specific effects, Cox models were fitted for the five pGRSs in the total cohort, and in the stratified *APP*dup and DS groups. We added the three different cohorts as covariates. In a subset of *APP*dup carriers, duplication length and individual genes located on chromosome 21 (duplicated in ≥ 5 carriers) were associated with AAO in separate Cox models. HRs and 95% CIs were reported. We controlled for the false discovery rate (FDR) to correct for multiple testing; *q* values < 0.05 were considered statistically significant.

## 3 RESULTS

### 3.1 Population and clinical characteristics

**FIGURE 1:**
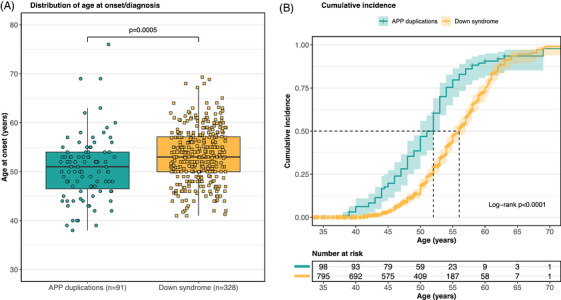
Distribution of AAO and cumulative incidence of AD stratified by *APP* duplications and Down syndrome. A, AAO distribution stratified by *APP* duplications and Down syndrome. The definition of AAO differs per cohort and includes the age at symptom onset, age at (prodromal) AD diagnosis, or age at first visit in case of established AD. B, Cumulative incidence of symptoms stratified by *APP* duplications (median AAO [95% confidence interval] = 52 years [50–53]) and Down syndrome (median AAO = 56 years [55–57]). AAO, age at onset; AD, Alzheimer's disease; *APP*, amyloid precursor protein.

**FIGURE 2:**
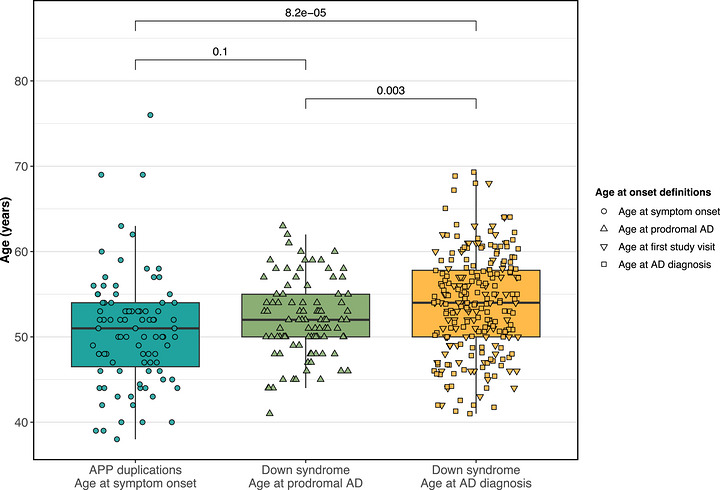
Distribution of age at symptom onset, prodromal AD, and dementia diagnosis. Boxes represent IQR, with the median shown as the horizontal line. The left boxplot shows the age at symptom onset distribution of *APP* duplication carriers (median 51 years [IQR 47–54]), the middle boxplot shows the age at prodromal AD diagnosis in Down syndrome (52 years [50–55]), and the right boxplot shows the age at dementia diagnosis in Down syndrome (54 years [50–58]). In this last group, we included both individuals with age at first study visit with dementia and individuals with clinical age at dementia diagnosis. AD, Alzheimer's disease; *APP*, amyloid precursor protein; IQR, interquartile range.

In total, 100 *APP*dup carriers were included from the Netherlands (*n =* 14), France (*n =* 46), Spain (*n =* 2), EADB (*n =* 6), DIAN (*n =* 8), and from literature (*n =* 24, Supplementary Results in supporting information). We included 957 individuals with DS from the DABNI cohort (*n =* 374), ACE (*n =* 12), and HUP (*n =* 571). Clinical characteristics are summarized in Table 1. The mean age at last visit among asymptomatic individuals was 42.4 years (SD ± 9.7), which did not differ between *APP*dup and DS (*P =* 0.43). A higher proportion of *APP*dup carriers was symptomatic for AD compared to individuals with DS (91% vs. 34%; *P <* 0.0001). The total cohort had a mean AAO of 52.8 ± 5.8 years, with AAO distribution shown in Figure 1. Mean AAO was earlier in *APP*dup carriers (mean AAO 50.7 ± 6.6 years) than individuals with DS (53.3 ± 5.5 years; *P =* 0.0005). However, AAO variability was similar between groups (Levene test: *F* = 1.12, *P =* 0.29), and AAO did not differ between *APP*dup carriers and DS individuals with an available age at prodromal AD diagnosis (*P =* 0.10; Figure 2). *APOE* genotype distributions were similar across groups (*P_APOE2_* = 0.80; *P_APOE4_* = 0.50), with most individuals homozygous for *APOE* ε3 (70%). There were no homozygous ε2 carriers and only ten homozygous ε4 carriers in DS (four asymptomatic and six symptomatic). Among *APP*dup carriers, duplication size ranged from 0.14 Mb to 18.50 Mb, encompassing between 1 and 65 genes.

**TABLE 1:** Demographic and clinical characteristics.

	Total cohort		Stratified groups		
	N	Total *N =* 1057^a^	*APP* duplications *N =* 100^a^	Down syndrome *N =* 957^a^	*p* value
**Age at last visit (asymptomatic)** ^b^	638	42.4 (9.7)	46.0 (13.1)	42.4 (9.6)	0.43^c^
**Age at onset (symptomatic)**	419	52.8 (5.8)	50.7 (6.6)	53.3 (5.5)	0.0005^c^
**Sex**	1051				0.17^d^
Male		549 (52%)	56 (60%)	493 (52%)	
Female		502 (48%)	38 (40%)	464 (48%)	
**Alzheimer's disease**	1057				<0.0001^d^
Symptomatic		419 (40%)	91 (91%)	328 (34%)	
Asymptomatic		638 (60%)	9 (9.0%)	629 (66%)	
***APOE***	1057				
*APOE* ε2 carrier		108 (10%)	9 (9.0%)	99 (10%)	0.80^d^
*APOE* ε4 carrier		212 (20%)	17 (17%)	195 (20%)	0.50^d^

^a^ Mean (SD), no. (%)

^b^ The age at last visit refers to the age at last visit of asymptomatic individuals only.

^c^ Welch two‐sample *t* test.

^d^ Pearson chi‐squared test. In the *APP* duplication group the asymptomatic individuals are from the Dutch (*n =* 2), French (*n =* 2), DIAN (*n =* 4) sites and from literature (*n =* 1). In the Down syndrome group, the asymptomatic individuals are from both the DABNI cohort (*n =* 205) and ACE/HUP (*n =* 424). Of the symptomatic individuals with DS in the DABNI cohort, 77 had established dementia before their first study visit.

Abbreviations: ACE/HUP, Ace Alzheimer Center Barcelona/Hospital Universitario de la Princesa; *APOE*, apolipoprotein E; *APP*, amyloid precursor protein; DABNI, Down Alzheimer's Barcelona Neuroimaging Initiative; DIAN, Dominantly Inherited Alzheimer Network; DS, Down syndrome; SD, standard deviation.

### 3.2 Associations of *APOE* and AD‐GRS with AAO

**FIGURE 3:**
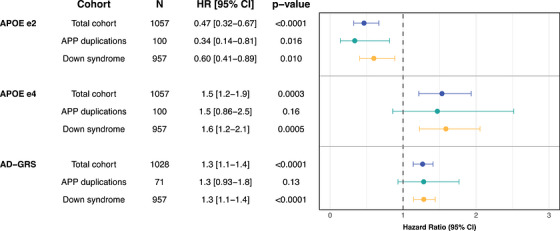
Cox proportional hazard model of *APOE* and AD genetic risk score associated with age at onset. The HRs for the *APOE* ε2 and ε4 correspond to the results from Model 1 (*APOE* ε2 + *APOE* ε4). The HRs for the AD‐GRS correspond to the results from Model 2 (*APOE* ε2 + *APOE* ε4 + AD‐GRS). The AD‐GRS included 77 single nucleotide polymorphisms associated with AD in which the *APOE* genotype was not included. AD, Alzheimer's disease; AD‐GRS, Alzheimer's disease genetic risk score; *APOE*, apolipoprotein E; *APP*, amyloid precursor protein; CI, confidence interval; HR, hazard ratio; IQR, interquartile range.

Figure 3 summarizes the Cox proportional hazard models. In the total cohort, *APOE* genotype was significantly associated with AAO. In Model 1, *APOE* ε2 delayed onset of AD (HR = 0.47 [0.32–0.67], *P <* 0.0001), whereas *APOE* ε4 accelerated onset (HR = 1.5 [1.2–1.9], *P =* 0.0003). In Model 2, the AD‐GRS was significantly associated with AAO (HR = 1.3 [1.1–1.4], *P <* 0.0001), indicating earlier onset with a higher AD‐GRS. No interaction between *APOE* ε2 or ε4 and the AD‐GRS was observed (*P_APOE2_* = 0.62;*P_APOE4_* = 0.36; Table S2 in supporting information).

Stratified analyses by *APP*dup and DS yielded consistent results. *APOE* ε2 delayed onset (HR_*APP*dup_ = 0.34 [0.14–0.81], *P =* 0.016; HR_DS_ = 0.60 [0.41–0.89], *P =* 0.010), while *APOE* ε4 accelerated onset (HR*APP*dup  = 1.5 [0.86–2.5], *P =* 0.16; HRDS = 1.6 [1.2–2.1], *P =* 0.0005). The effect of AD‐GRS was similar in *APP*dup (HR = 1.3 [0.93–1.8], *P =* 0.13) and DS (HR = 1.3 [1.1–1.4], *P <* 0.0001). In stratified analyses of the DS cohorts, the effect of the *APOE* ε2 was weaker in the DABNI cohort (Figure S1 in supporting information). The meta‐analysis of the single variant analyses underlying the AD‐GRS showed that the association was not driven by a single variant (Table S3 in supporting information), but by small effects of all variants (genomic inflation factor = 1.45; Figure S2 in supporting information).

### 3.3 Median AAO prediction

**FIGURE 4:**
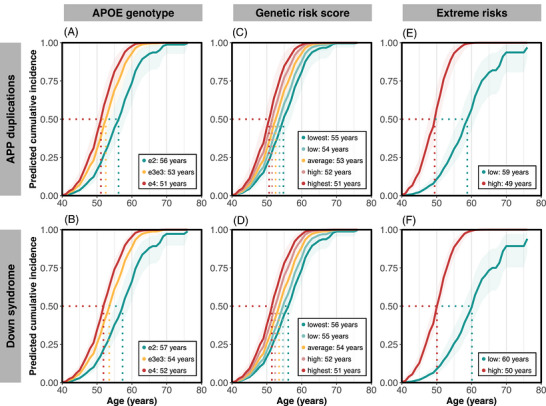
Predicted cumulative incidence for *APOE* genotypes, genetic risk scores, and extremes stratified by *APP* duplications and Down syndrome. These figures show the predicted cumulative incidence curves stratified by *APP* duplications and Down syndrome. In (A) and (B), the predicted curves for the different *APOE* genotypes are shown. (C) and (D) show the predicted curves for the different scores for the AD‐GRS, in which the lowest risk corresponds with –2 SDs below the mean AD‐GRS, the low risk with –1 SD, average with the mean AD‐GRS, high risk with +1 SD, and highest risk with +2 SD. The AD‐GRS includes 77 single nucleotide polymorphisms (*APOE* is excluded). In (E) and (F), the predicted cumulative incidence curves of the two most extreme risk groups are shown. The lowest risk includes *APOE ε*2 and the lowest AD‐GRS (–2 SD), the highest risk includes *APOE ε*4 and the highest AD‐GRS (+2 SD). AD‐GRS, Alzheimer's disease genetic risk score, *APOE*, apolipoprotein E; *APP*, amyloid precursor protein; SD, standard deviation.

Given differences in AAO between *APP*dup and DS, we report stratified results that showed similar overall patterns in both groups (Figure 4 and Table S4 in supporting information). In *APP*dup, predicted median AAO was 51.1 years [49.8–52.3] in *APOE* ε4, 52.5 years [51.3–53.9] in *APOE* ε3/ε3, and 56.2 years [53.9–58.2] in *APOE* ε2. Corresponding AAOs in DS were 51.8 years [51.1–52.8], 53.5 years [52.6–54.3], and 57.3 years [55.1–59.4], respectively. For the AD‐GRS, predicted median AAO in *APP*dup ranged from 50.6 years [49.3–52.0] at the highest AD‐GRS (+2 SD) to 54.7 years [53.2–56.9] at the lowest AD‐GRS (–2 SD). In DS, these AAOs ranged from 51.4 years [50.4–52.5] at the highest to 56.1 years [54.5–57.5] at the lowest AD‐GRS. Combining *APOE* and AD‐GRS, predicted median AAO differed by 9.4 years between the genetic extremes in *APP*dup (highest risk: 49.4 years [47.7–50.7]; lowest risk: 58.8 years [56.5–61.1]). In DS, the corresponding difference was 10 years (highest risk: 50.1 years [49.2–51.1]; lowest risk: 60.1 years [57.6–62.4]). Cumulative incidence curves stratified by *APOE* are presented in Figure S3 in supporting information and corresponding predicted AAOs in Table S5 in supporting information. Within each *APOE* genotype, higher AD‐GRS values were consistently associated with earlier predicted median AAO.

### 3.4 Pathway‐specific GRS analyses

**FIGURE 5:**
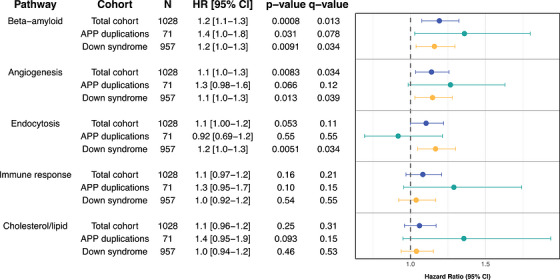
Cox proportional hazard models of pathway‐specific genetic risk scores with age at onset. The amyloid beta pGRS included 60 SNPs, the angiogenesis pGRS 41 SNPs, the endocytosis pGRS 34 SNPs, the immune response pGRS 42 SNPs, and the cholesterol/lipid pGRS 39 SNPs. The *APOE* genotype was not included in these pGRSs. *APOE*, apolipoprotein E; *APP*, amyloid precursor protein; CI, confidence interval; HR, hazard ratio; pGRS, pathway genetic risk score; SNP, single nucleotide polymorphisms.

Pathway‐specific GRS analyses are shown in Figure 5, and correlations between each pGRS are presented in Figure S4 in supporting information. In the total cohort, the Aβ pGRS (HR = 1.2 [1.1–1.3], *q =* 0.013) and angiogenesis pGRS (HR = 1.1 [1.0–1.3], *q =* 0.034) were associated with AAO. The pGRS for endocytosis (HR = 1.1 [1.0–1.2], *q =* 0.11), immune response (HR = 1.1 [0.97–1.2], *q =* 0.21), and cholesterol/lipids (HR = 1.1 [0.96–1.2], *q =* 0.31) did not reach statistical significance. In DS, the endocytosis pGRS was associated with AAO (HR = 1.2 [1.0–1.3], *q =* 0.034). The effects in *APP*dup and DS were comparable, albeit less significant in *APP*dup, consistent with smaller sample size.

### 3.5 Duplication length and chromosome 21 gene content

Finally, in a selection of *APP*dup carriers (*n =* 92), we did not find an association of duplication length with AAO (HR = 1.00 [0.96–1.05], *P =* 0.95). We then assessed the association of 23 individual genes from chromosome 21 with AAO (located between chr21:13,609,777–29,536,93; GRCh38/hg38). Duplication of none of these genes were significantly associated with AAO (Table S6 in supporting information).

## 4 DISCUSSION

We investigated whether established genetic risk factors for sporadic AD modify AAO in individuals with an extra copy of *APP*, either in ADAD or as part of trisomy 21. To our knowledge, this is the first study to demonstrate robust effects of both *APOE* and AD‐GRS on AAO in *APP*dup carriers and the largest study of genetic modifiers in individuals with an extra copy of *APP*. In both *APP*dup and DS, onset of AD was accelerated by *APOE* ε4 and a high AD‐GRS, and delayed by *APOE* ε2 and a low AD‐GRS. The effects of *APOE* and AD‐GRS were independent and, at the extremes of genetic risk, amounted to a 9‐ to 10‐year difference in AAO. These modifying effects were consistent across *APP*dup carriers and those with DS, and were not driven by any single variant. Pathway analyses instead pointed to variants related to the Aβ and angiogenesis pathways as having the strongest influence on AAO, suggesting that these pathways may be particularly important in shaping the effects of *APP* overexpression.

Our findings show that *APOE* ε4 accelerates and *APOE* ε2 delays AAO, consistent with sporadic AD and with several studies in ADAD and DS.^15^, ^16^, ^17^, ^18^, ^20^, ^21^, ^34^, ^35^ Across studies, effect directions are largely consistent, although effect sizes and significance vary and one report described an association in the opposite direction.^18^ Studies focusing on specific pathogenic variants often report clearer effects, whereas analyses pooling multiple ADAD variants tend to show smaller or non‐significant effects, suggesting variant‐dependent modification.^17^, ^19^, ^21^, ^25^ In DS, *APOE* associations with clinical outcomes have not been consistently observed, including no association with age at memory change in a previous smaller study.^22^ In our study, the *APOE* ε4 effect did not reach statistical significance in the smallest group (*APP*dup), although effect sizes were comparable to those in DS. Notably, *APOE* ε2 showed a larger delaying effect than *APOE* ε4's accelerating effect. We speculate that in individuals with an extra *APP* copy, *APOE* ε2 may confer relatively stronger protection by enhancing amyloid clearance, whereas the already elevated amyloid burden may constrain the additional acceleration attributable to *APOE* ε4. Overall, these findings support *APOE* as a modifier of AAO in the context of an extra *APP* copy.

The AD‐GRS was also associated with AAO in individuals with an extra *APP* copy: higher scores accelerated AAO, and lower scores delayed AAO. Again, these findings are in line with literature on sporadic AD.^35^ The AD‐GRS has not previously been evaluated in *APP*dup, but prior work linked the AD‐GRS to AD biomarkers in ADAD,^24^ and to memory performance and CSF profiles in DS.^22^ In our *APP*dup sample, the direction and magnitude of the effect were consistent, but did not reach statistical significance due to the smaller sample size. Together, these studies and our results support the notion that polygenic AD susceptibility contributes to variation in AAO in individuals with an extra copy of *APP*.

When considered jointly, *APOE* and the AD‐GRS corresponded to an estimated 9‐ to 10‐year difference in AAO between individuals at the lowest and highest genetic risk. In sporadic AD, the AAO difference between these extreme risks was larger (≈ 20 years).^35^ However, AD onset in individuals with an extra *APP* copy occurs within a narrower and earlier range (≈ 40–60 years) than in sporadic AD (≈ 60–100 years), making a 9‐ to 10‐year shift substantial and relatively comparable to sporadic AD. Several non‐genetic risk factors have been proposed in DS, including sensory impairment, sleep disturbances, and mental health problems, but evidence is mixed and inconsistent across studies.^20^, ^36^ By contrast, no non‐genetic modifiers of AAO are known for *APP*dup.^14^ Thus, *APOE* and the AD‐GRS remain the only reproducible predictors of AAO in individuals with an extra copy of *APP*.

Pathway‐specific analyses implicated the Aβ and angiogenesis pathways as modifiers of AAO. Although not significant in *APP*dup (both *q =* 0.16), effect sizes were similar in DS, suggesting a consistent pattern across groups. This supports a prominent role for amyloid processing and vascular biology in *APP*‐driven AD, whereas the immune response pathway showed only a small effect despite its major contribution in sporadic AD.^26^ In the context of *APP* overexpression, common variants affecting *APP* processing, amyloid clearance, and amyloid‐related vascular pathology may have amplified effects on AAO even when baseline amyloid production is high.^1^ We also observed an association of the endocytosis pathway with AAO in DS but not in *APP*dup, possibly because endosomal function is already disrupted in DS and therefore more sensitive to modification by common genetic variation.^37^ We urge consideration of these observations when developing interventions aimed at postponing AAO of AD in individuals with an extra copy of *APP*.

To explore whether additional genes on chromosome 21 contribute to delayed onset in DS, we examined duplication length and gene content in *APP*dup carriers. Consistent with previous findings,^14^ no duplicated genes were associated with AAO, suggesting that variation within the smaller duplicated regions observed in *APP*dup carriers does not substantially modify disease onset. In contrast, the broader trisomy in DS may include additional chromosome 21 genes that could influence AAO. Experimental studies have shown that triplication of chromosome 21 regions beyond *APP* can modulate amyloid pathology and survival in *APP*‐overexpressing mouse models, implicating genes such as *DYRK1A*, *BACE2*, and *SYNJ1* as potential modifiers.^38^ These findings raise the possibility that genes outside the *APP*dup regions contribute to disease heterogeneity in DS, although their effects on AAO remain to be established and are difficult to disentangle from the effect of intellectual deficits on diagnosing AD in DS.^39^

A major strength of this study is the large, combined cohort of individuals with an extra copy of *APP*. The inclusion of asymptomatic DS individuals is important as excluding unaffected individuals may reduce the power to detect genetic modifiers that delay AAO. A limitation is the different AAO definition across cohorts, constraining direct comparisons of mean onset ages between *APP*dup and DS. In *APP*dup, AAO reflected retrospective age at first symptoms, whereas in DS, AAO was defined as age at (prodromal) AD diagnosis or age at first visit with already established dementia. By definition, symptom onset precedes clinical AD diagnosis, and in DS, clinical recognition is delayed due to pre‐existing intellectual disability, frequent comorbidities, and atypical clinical presentations.^7^ These factors likely contributed to an underestimation of true symptom onset in DS. Importantly, the absolute difference in mean AAO between *APP*dup and DS was small (≈ 2.5 years). We therefore interpret the younger mean AAO observed in *APP*dup primarily as a definitional rather than a true biological difference. This interpretation is supported by the observation that AAO in individuals with DS who had a recorded age at prodromal AD diagnosis was comparable to the AAO observed in *APP*dup carriers. Moreover, the variability in onset was similar between *APP*dup and DS, consistent with a previous meta‐analysis showing comparable AAO variability in DS‐AD and ADAD.^11^ Future studies could also investigate whether *APOE* and AD‐GRS influence the timing of amyloid positron emission tomography positivity and other AD‐related biomarker changes. However, such analyses may be more feasible in DS than in *APP*dup, as individuals with DS are often followed longitudinally before symptom onset, whereas *APP*dup carriers are typically identified after symptom onset or through a known family history.

In conclusion, our findings suggest that incorporating the *APOE* genotype and AD‐GRS could improve prediction of AAO in these individuals. Because these genetic factors likely act through specific biological pathways, they may also influence response to pathway‐targeted interventions. Our pathway analyses highlight the roles of Aβ, angiogenesis, and endocytosis mechanisms in *APP*‐driven AD, implying that the pathways implicated in sporadic and genetically determined AD overlap. Future (pharmacological) studies in pre‐symptomatic individuals with an extra *APP* copy should consider underlying genetic risk for sporadic AD, as these variants may enhance or diminish treatment efficacy.

## AUTHOR CONTRIBUTIONS

Joan Groeneveld, Juan Fortea, Lisa Vermunt, Gael Nicolas, Oriol Dols‐Icardo, Olivia Belbin, and Sven J. van der Lee conceptualized and designed the study. Joan Groeneveld, Danna Perlaza, Clàudia Olivé, Lou Grangeon, Niccolo Tesi, Chenyang Jiang, Itziar de Rojas, David Wallon, Stéphane Rousseau, Diego Real de Asúa, Fernando Moldenhauer, Ruihao Mu, Kévin Cassinari, Aline Zarea, Joaquim Aumatell Escabias, Marc Hulsman, Alan E. Renton, Alison M. Goate, and Gael Nicolas had a major role in the acquisition of data. Marta Rovira, Joaquim Aumatell Escabias, Marc Hulsman, and Clàudia Olivé had a major role in cleaning and computing the data. Joan Groeneveld, Danna Perlaza, and Clàudia Olivé were responsible for the statistical analysis. Joan Groeneveld, Danna Perlaza, Clàudia Olivé, Maria Victoria Fernandez, Olivia Belbin, Gael Nicolas, Oriol Dols‐Icardo, and Sven J. van der Lee interpreted the data. Joan Groeneveld and Danna Perlaza drafted the manuscript. Clàudia Olivé, Niccolo Tesi, Chenyang Jiang, Itziar de Rojas, David Wallon, Stéphane Rousseau, Marta Rovira, Jean‐Charles Lambert, Yolande A.L. Pijnenburg, Everard G. B. Vijverberg, Johannes Levin, Mathias Jucker, Eric McDade, Juan Fortea, Henne Holstege, Flora H. Duits, Lisa Vermunt, Maulikkumar Patel, Matthew Johnson, Alan E. Renton, Alison M. Goate, Carlos Cruchaga, Cyril Pottier, Maria Victoria Fernandez, Olivia Belbin, Gael Nicolas, Oriol Dols‐Icardo, and Sven J. van der Lee carefully read the manuscript. Joan Groeneveld, Danna Perlaza, Clàudia Olivé, David Wallon, Alan E. Renton, Carlos Cruchaga, Olivia Belbin, Maria Victoria Fernandez, Olivia Belbin, Gael Nicolas, Oriol Dols‐Icardo, and Sven J. van der Lee revised the manuscript.

## CONFLICT OF INTEREST STATEMENT

Joan Groeneveld, Danna Perlaza, Clàudia Olivé, Lou Grangeon, Niccolo Tesi, Aude Nicolas, Chenyang Jiang, Itziar de Rojas, David Wallon, Stéphane Rousseau, Marta Rovira, Diego Real de Asúa, Fernando Moldenhauer, Ruihao Mu, Kévin Cassinari, Aline Zarea, Joaquim Aumatell Escabias, Jean‐Charles Lambert, Yolande A. L. Pijnenburg, Marc Hulsman, Everard G. B. Vijverberg, Mathias Jucker, Henne Holstege, Flora H. Duits, Lisa Vermunt, Maulikkumar Patel, Matthew Johnson, Alan E. Renton, Alison M. Goate, Carlos Cruchaga, Cyril Pottier, Maria Victoria Fernandez, Gael Nicolas, Oriol Dols‐Icardo, and Sven J. van der Lee have nothing to disclose. Juan Fortea reports serving on the advisory boards, adjudication committees, or speaker honoraria for AC Immune, Adamed, Alzheon, Biogen, Eisai, Esteve, Fujirebio, Ionis, Laboratorios Carnot, Life Molecular Imaging, Lilly, Novo Nordisk, Perha, Roche, and Zambón. Juan Fortea reports holding a patent for markers of synaptopathy in neurodegenerative disease (licensed to ADx, WO2019175379). Olivia Belbin reports holding a patent for markers of synaptopathy in neurodegenerative disease (licensed to ADx NeuroSciences N.V., WO2019175379 Markers of synaptopathy in neurodegenerative diseases). No other competing interests were reported. Johannes Levin reports speaker fees from Bayer Vital, Biogen, EISAI, Lilly, TEVA, Bial, Zambon, Esteve, Merck, and Roche; consulting fees from Axon Neuroscience, EISAI, Alnylam, and Biogen; author fees from Thieme medical publishers and W. Kohlhammer GmbH medical publishers; and is inventor in a patent “Oral Phenylbutyrate for Treatment of Human 4‐Repeat Tauopathies” (PCT/EP2024/053388) filed by LMU Munich. In addition, he reports compensation for serving as chief medical officer for MODAG GmbH, is beneficiary of the phantom share program of MODAG GmbH, and is inventor in a patent “Pharmaceutical Composition and Methods of Use” (EP 22 159 408.8) filed by MODAG GmbH, all activities outside the submitted work. Eric McDade has received research support from the National Institutes of Health, the National Institute on Aging (R01AG068319, U19AG032438, U01AG059798), Alzheimer's Association (DIAN‐TU‐PP‐22‐872356, DIAN‐TU‐TAU‐21‐822987, DIAN‐TU‐OLE‐21‐725093), GHR Foundation, an anonymous organization, Knight Family, and the DIAN‐TU Pharma Consortium (Biogen, Eisai, Eli Lilly and Company/Avid Radiopharmaceuticals, F. Hoffman‐La Roche/Genentech, and Janssen). He is a co‐inventor of the “Methods of diagnosing AD with phosphorylation changes” technology licensed by Washington University to C2N Diagnostics, US Patent 11,085,935 B2, Aug 10, 2021 (Bateman, Barthelemy, McDade, Li). Washington University also holds 5% equity in C2N. Through these relationships, Washington University and Eric McDade are entitled to receive royalties from the license agreement with C2N. He is co‐author of the Alzheimer Association's 2024 Alzheimer's disease updated diagnostic criteria. He has received travel support from the Alzheimer's Association for work related to the development of the document. He has served as a consultant, a member of advisory boards, or a DSMB member for Eli Lilly, Alector, Alzamend, Sanofi, AstraZeneca, Hoffman La‐Roche, and Merck. Author disclosures are available in the Supporting Information.

## DATA SHARING

Individual de‐identified participant and summary statistics of genetic data not published in this article will be made available upon reasonable request to any qualified investigator by contacting Dr. S.J. van der Lee (s.j.vanderlee@amsterdamumc.nl). Requests regarding data from the DABNI Cohort will be assessed by Dr. Juan Fortea and Dr. Oriol Dols‐Icardo. The raw data from DIAN Obs are available under restricted access to maintain individual and family confidentiality. These samples contain rare disease‐causing variants that could be used to identify the participating individuals and families. Access can be obtained by request through the online resource request system on the DIAN Website: https://dian.wustl.edu/.

## CONSENT STATEMENT

All participants or their legal representatives provided written informed consent, including consent for genetic analyses.

## Supporting information




**Supporting Information**: alz71738‐sup‐0001‐SuppMat.pdf

**Supporting Information**: alz71738‐sup‐0002‐SuppMat.pdf

## Appendix A Collaborators list

### A.1

Dominantly Inherited Alzheimer Network

**Table:**

Last Name	First Name	Affiliation	Email Address
Bateman	Randall	Washington University School of Medicine in St. Louis	batemanr@wustl.edu
Daniels	Alisha J.	Washington University in St. Louis	alisha.daniels@wustl.edu
Courtney	Laura	Washington University in St. Louis	lcourtne@wustl.edu
Ziegemeier	Angela	Washington University in St. Louis	zangela@wustl.edu
Skrbec	Karina	Washington University in St. Louis	skrbec@wustl.edu
Hellm	Cortaiga	Washington University in St. Louis	cortaiga.hellm@wustl.edu
Martin	Mariana	Washington University in St. Louis	marianam@wustl.edu
Ziegemeier	Ellen	Washington University in St. Louis	eziegem@wustl.edu
Bartzel	Jamie	Washington University in St. Louis	bartzel@wustl.edu
McDade	Eric	Washington University School of Medicine in St. Louis, Department of Neurology	ericmcdade@wustl.edu
Llibre‐Guerra	Jorge J.	Dominantly Inherited Alzheimer's Network Department of Neurology, Washington University School of Medicine in St. Louis	jllibre-guerra@wustl.edu
Supnet‐Bell	Charlene	Washington University in St. Louis, School of Medicine, Department of Neurology	supnet@wustl.edu
Xiong	Chengie	Washington University in St. Louis, School of Medicine	chengjie@wuslt.edu
Xu	Xiong	Washington University in St. Louis, School of Medicine	xxu@wustl.edu
Lu	Ruijin	Washington University in St. Louis, School of Medicine	r.lu@wustl.edu
Wang	Guoqiao	Washington University in St. Louis, School of Medicine	guoqiao@wustl.edu
Li	Yan	Washington University in St. Louis, School of Medicine	Yanli833@wustl.edu
Nie	Yuzheng	Washington University in St. Louis, School of Medicine	n.yuzheng@wustl.edu
Gremminger	Emily	Washington University in St. Louis, School of Medicine	egremminger@wustl.edu
Arora	Jyoti	Washington University in St. Louis, School of Medicine	j.arora@wustl.edu
Perrin	Richard J.	Department of Pathology and Immunology, Department of Neurology, Knight Alzheimer Disease Research Center, Washington University School of Medicine, Saint Louis, MO, USA,	rperrin@wustl.edu
Franklin	Erin E.	Department of Pathology and Immunology, Washington University in St. Louis	efranklin@wustl.edu
Ibanez	Laura	Washington University in St. Louis, Departments of Psychiatry & Neurology; NeuroGenomics and Informatics Center	ibanezl@wustl.edu
Jerome	Gina	Washington University in St. Louis, School of Medicine, Department of Psychiatry	ginajerome@wustl.edu
Stauber	Jennifer	Washington University in St. Louis, School of Medicine, Department of Psychiatry	jenniferstauber@wustl.edu
Baker	Bryce	Washington University in St. Louis, School of Medicine, Department of Psychiatry	bbaker26@wustl.edu
Minton	Matthew	Washington University in St. Louis, School of Medicine, Department of Psychiatry	mminton@wustl.edu
Preminger	Sam	Washington University in St. Louis, School of Medicine, Department of Psychiatry	psam@wustl.edu
Cruchaga	Carlos	1. Department of Psychiatry, Washington University School of Medicine, St. Louis, MO, USA 2. NeuroGenomics and Informatics Center, Washington University School of Medicine, St. Louis, MO, USA	cruchagac@wustl.edu
Goate	Alison M.	1.Jean C. & James W. Crystal Professor and Chair 2. Director, Ronald M. Loeb Center for Alzheimer's disease 3. Dept. of Genetics & Genomic Sciences, Icahn Genomics Institute 4. Icahn School of Medicine at Mount Sinai, New York, NY	alison.goate@mssm.edu
Renton	Alan E.	Ronald M. Loeb Center for Alzheimer's Disease, Dept of Genetics and Genomic Sciences, Icahn School of Medicine at Mount Sinai, New York, NY, USA	alan.renton@mssm.edu
Picarello	Danielle M.	Ronald M. Loeb Center for Alzheimer's Disease, Dept of Genetics and Genomic Sciences and Nash Family Dept of Neuroscience, Icahn School of Medicine at Mount Sinai, New York, NY, USA	danielle.picarello@mssm.edu
Fulton‐Howard	Brian	Ronald M. Loeb Center for Alzheimer's Disease, Dept of Genetics and Genomic Sciences and Nash Family Dept of Neuroscience, Icahn School of Medicine at Mount Sinai, New York, NY, USA	
Benzinger	Tammie L.S.	Washington University in St. Louis	benzingert@wustl.edu
Gordon	Brian A.	Washington University in St. Louis	bagordon@wustl.edu
Banks	Jessica	Washington University in St. Louis	jessica.banks@wustl.edu
Hornbeck	Russ	Washington University in St. Louis	russ@wustl.edu
Chen	Allison	Washington University in St. Louis	
Chen	Charles	Washington University in St. Louis	cdchen@wustl.edu
Flores	Shaney	Washington University in St. Louis	sflores@wustl.edu
Goyal	Manu	Washington University in St. Louis	goyalm@wustl.edu
Joseph‐Mathurin	Nelly	Washington University in St. Louis	n.joseph@wustl.edu
Jackson	Kelley	Washington University in St. Louis	kelleyj@wustl.edu
Keefe	Sarah	Washington University in St. Louis	sarahkeefe@wustl.edu
Koudelis	Deborah	Washington University in St. Louis	delanod@wustl.edu
Massoumzadeh	Parinaz	Washington University in St. Louis	massoumzadehp@wustl.edu
McKay	Nicole	Washington University in St. Louis	n.mckay@wustl.edu
Wang	Qing	Washington University in St. Louis	wangqing@wustl.edu
Sabaredzovic	Edita	Washington University in St. Louis	edita@wustl.edu
Scott	Jalen	Washington University in St. Louis	jalenscott@wustl.edu
Simmons	Ashlee	Washington University in St. Louis	ashlees@wustl.edu
Rizzo	Jacqueline	Washington University in St. Louis	jrizzo@wustl.edu
Vlassenko	Andrei	Washington University in St. Louis	andreigvlassenko@wustl.edu
Wang	Yong	Washington University in St. Louis	wangyong@wustl.edu
Smith	Thomas	Washington University in St. Louis	smiththomas@wustl.edu
Murphy	Mei	Washington University in St. Louis	m.e.murphy@wustl.edu
Ances	Beau	Washington University in St. Louis	bances@wustl.edu
Dombrowski	Kaitlyn	Washington University in St. Louis	kdombrowski@wustl.edu
Hoagey	David	Washington University in St. Louis	hoagey@wustl.edu
Millar	Peter	Washington University in St. Louis	pmillar@wustl.edu
Powles	Savannah	Washington University in St. Louis	savannahpowles@wustl.edu
Melson	Griffin	Washington University in St. Louis	griffinm@wustl.edu
Hassenstab	Jason	Norman J. Stupp Professor of Neurology; Professor of Psychological & Brain Sciences Washington University in St. Louis	hassenstabj@wustl.edu
Smith	Jennifer	Department of Neurology, Washington University in St. Louis	smith.jennifer@wustl.edu
Stout	Sarah	Department of Neurology, Washington University in St. Louis	shstout@wustl.edu
Vila‐Castelar	Clara	Department of Neurology, Washington University in St. Louis	clarav@wustl.edu
Frank	Colleen	Department of Neurology, Washington University in St. Louis	frankc@wustl.edu
Aschenbrenner	Andrew J.	Assistant Professor of Neurology	aaschenbrenner@kumc.edu
Karch	Celeste M.	Department of Psychiatry, Washington University in St Louis	karchc@wustl.edu
Marsh	Jacob	DIAN Fibroblast and Stem Cell Bank, Washington University in St. Louis	jacobmarsh@wustl.edu
Morris	John C.	Washington University in St. Louis, Department of Neurology and the Knight Alzheimer Disease Research Center	jcmorris@wustl.edu
Holtzman	David M.	Department of Neurology, Knight Alzheimer's Disease Research Center, Hope Center for Neurological Disorders, Washington University in St. Louis	holtzman@wustl.edu
Barthélemy	Nicolas R.	1. Washington University in St. Louis, School of Medicine, Department of Neurology 2. Tracy Family Stable Isotope Labeling Quantitation (SILQ) Center, Washington University School of Medicine, St. Louis, MO, USA	barthelemy.nicolas@wustl.edu
Xu	Jinbin	Washington University in St. Louis	jinbinxu@wustl.edu
Berman	Sarah B.	University of Pittsburgh, Departments of Medicine and Neurology	bermans@upmc.edu
Nadkarni	Neelesh	University of Pittsburgh, Departments of Medicine	nadkarnink@upmc.edu
Ikonomovic	Snezana	University of Pittsburgh, Department of Neurology	ikonomovics@upmc.edu
Day	Gregory S.	Mayo Clinic in Florida; Jacksonville, FL, USA, Department of Neurology	Day.Gregory@mayo.edu
Lachner	Christian	Mayo Clinic in Florida; Jacksonville, FL, USA, Department of Neurology and Departments of Psychiatry and Psychology	lachner.christian@mayo.edu
Farlow	Martin	1. Indiana School of Medicine 2. Indiana University Health	mfarlow@iu.edu
Chhatwal	Jasmeer P.	Massachusetts General Hospital, Brigham and Women's Hospital, Harvard Medical School	Chhatwal.Jasmeer@mgh.harvard.edu
Pinilla	Valentina	Massachusetts General Hospital, Brigham and Women's Hospital, Harvard Medical School	
Maa	Courtney	Massachusetts General Hospital, Brigham and Women's Hospital, Harvard Medical School	cmaa@mgh.harvard.edu
Ikeuchi	Takeshi	Brain Research Institute, Niigata University	ikeuchi@bri.niigata-u.ac.jp
Ishiguro	Takanobu	Brain Research Institute, Niigata University	tishiguro@bri.niigata-u.ac.jp
Aoyama	Azusa	Brain Research Institute, Niigata University	a.aoyama.bri@niigata-u.ac.jp
Ishii	Kenji	Tokyo Metropolitan Institute of Gerontology	ishii@pet.tmig.or.jp
Senda	Michio	Kobe City Medical Center General Hospital	michio_senda@kcho.jp
Niimi	Yoshiki	Unit for Early and Exploratory Clinical Development, The University of Tokyo Hospital	niimiy-crc-@h.u-tokuo.ac.jp
Huey	Edward D.	Department of Psychiatry and Human Behavior, Alpert Medical School, Brown University	EHuey@Butler.org
Bodge	Courtney	Memory and Aging Program, Butler Hospital,	cbodge@butler.org
Salloway	Stephen	Memory and Aging Program, Butler Hospital, Departments of Psychiatry and Human Behavior and Neurology, Alpert Medical School, Brown University	SSalloway@butler.org
Devenney	Emma	1. Neuroscience Research Australia, Sydney NSW 2031 Australia 2. School of Clinical Medicine, University of New South Wales, Sydney NSW 2052 Australia	e.devenney@neura.edu.au
Schofield	Peter R.	1. Neuroscience Research Australia, Sydney NSW 2031 Australia 2. School of Biomedical Sciences, University of New South Wales, Sydney NSW 2052 Australia	p.schofield@neura.edu.au
Brooks	William S.	1. Neuroscience Research Australia, Sydney NSW 2031 Australia; and 2. School of Clinical Medicine, University of New South Wales, Sydney NSW 2052 Australia	w.brooks@neura.edu.au
Bechara	Jacob A.	Neuroscience Research Australia, Sydney NSW 2031 Australia	j.bechara@neura.edu.au
Martins	Ralph N.	1. Centre of Excellence for Alzheimer's Disease Research and Care, School of Medical and Health Sciences, Edith Cowan University, Joondalup, Western Australia, Australia. 2. Alzheimer's Research Australia, Ralph and Patricia Sarich Neuroscience Research Institute, Nedlands, Western Australia, Australia. 3.Department of Biomedical Sciences, Macquarie University, Sydney, New South Wales, Australia.	r.martins@ecu.edu.au
Sohrabi	Hamid R.	1. Alzheimer's Research Australia, Ralph and Patricia Sarich Neuroscience Research Institute, Nedlands, Western Australia, Australia. 2. Centre for Healthy Ageing, Health Futures Institute, Murdoch University, Murdoch, Western Australia, Australia. 3. School of Psychology, Murdoch University, Murdoch, Western Australia, Australia.	Hamid.Sohrabi@murdoch.edu.au
Taddei	Kevin	1. Centre of Excellence for Alzheimer's Disease Research and Care, School of Medical and Health Sciences, Edith Cowan University, Joondalup, Western Australia, Australia. 2. Alzheimer's Research Australia, Ralph and Patricia Sarich Neuroscience Research Institute, Nedlands, Western Australia, Australia.	k.taddei@ecu.edu.au
Gardener	Samanatha L.		s.gardener@ecu.edu.au
Fox	Nick C.	1. Dementia Research Centre, UCL Queen Square Institute of Neurology, London, United Kingdom 2. UK Dementia Research Institute at UCL, London, United Kingdom	n.fox@ucl.ac.uk
Cash	David M.	1. Dementia Research Centre, UCL Queen Square Institute of Neurology, London, United Kingdom 2. UK Dementia Research Institute at UCL, London, United Kingdom	d.cash@ucl.ac.uk
Ryan	Natalie S.	1. Dementia Research Centre, UCL Queen Square Institute of Neurology, London, United Kingdom 2. UK Dementia Research Institute at UCL, London, United Kingdom	natalie.ryan@ucl.ac.uk
Jucker	Mathias	1. German Center for Neurodegenerative Diseases (DZNE), Tübingen, Germany 2. Hertie‐Institute for Clinical Brain Research, University of Tübingen, Tübingen, Germany	Mathias.Jucker@uni-tuebingen.de
Laske	Christoph	1.German Center for Neurodegenerative Diseases (DZNE), Tübingen, Germany 2. Hertie‐Institute for Clinical Brain Research, University of Tübingen, Tübingen, Germany 3. Department of Psychiatry and Psychotherapy, University of Tübingen, Tübingen, Germany	christoph.laske@med.uni-tuebingen.de
Forkavets	Oksana	1. German Center for Neurodegenerative Diseases (DZNE), Tübingen, Germany 2. Hertie‐Institute for Clinical Brain Research, University of Tübingen, Tübingen, Germany	
Spring	Beatrice	1. German Center for Neurodegenerative Diseases (DZNE), Tübingen, Germany 2. Hertie‐Institute for Clinical Brain Research, University of Tübingen, Tübingen, Germany	
Graber‐Sultan	Susanne	1. German Center for Neurodegenerative Diseases (DZNE), Tübingen, Germany 2. Hertie‐Institute for Clinical Brain Research, University of Tübingen, Tübingen, Germany	susanne.graeber-sultan@dzne.de
la Fougère	Christian	1. German Center for Neurodegenerative Diseases (DZNE), Tübingen, Germany 2. Department of Nuclear Medicine and Clinical Molecular Imaging, University of Tübingen, Tübingen, Germany	
Reischl	Gerald	1. German Center for Neurodegenerative Diseases (DZNE), Tübingen, Germany 2. Werner Siemens Imaging Center, Department of Preclinical Imaging and Radiopharmacy, University of Tübingen, Tübingen, Germany	
Obermueller	Ulrike	1. German Center for Neurodegenerative Diseases (DZNE), Tübingen, Germany 2. Hertie‐Institute for Clinical Brain Research, University of Tübingen, Tübingen, Germany	ulrike.obermueller@klinikum.uni-tuebingen.de
Levin	Johannes	1. German Center for Neurodegenerative Diseases, site Munich; 2. Department of Neurology, Ludwig‐Maximilians‐Universität München, Munich, Germany; 3. Munich Cluster for Systems Neurology (SyNergy), Munich, Germany	Johannes.Levin@med.uni‐muenchen.de
Rude	Ilona	1. German Center for Neurodegenerative Diseases, site Munich	ilona.ruge@dzne.de
Vöglein	Jonathan	1. Department of Neurology, LMU University Hospital, LMU Munich, Munich, Germany 2. German Center for Neurodegenerative Diseases (DZNE), Munich, Germany 3.Munich Cluster for Systems Neurology (SyNergy, Munich, Germany	Jonathan.Voeglein@med.uni-muenchen.de
Lee	Jae‐Hong	Asan Medical Center, Department of Neurology, Seoul, Republic of Korea	jhlee@amc.seoul.kr
Roh	Jee Hoon	1. Korea University Anam Hospital, Department of Neurology, Seoul, Republic of Korea 2. Korea University College of Medicine, Department of Physiology and Department of Biomedical Sciences, Seoul, Republic of Korea	alzheimer@naver.com
Allegri	Ricardo F.	Instituto Neurológico Fleni, Buenos Aires, Argentina	rallegri@fleni.org.ar
Chrem Mendez	Patricio	Instituto Neurológico Fleni, Buenos Aires, Argentina	pchremmendez@fleni.org.ar
Surace	Ezequiel	Instituto Neurológico Fleni, Buenos Aires, Argentina, Department of Molecular Biology and Neuropathology,	esurace@hotmail.com
Vigo	Gabriela	Instituto Neurológico Fleni, Buenos Aires, Argentina	vigogab@hotmail.com
Aguillon	David	Grupo de Neurociencias de Antioquia (GNA), Facultad de Medicina, Universidad de Antioquia, Medellín, Colombia.	david.aguillon@gna.org.co
Guerrero	Alejandro	Grupo de Neurociencias de Antioquia (GNA), Facultad de Medicina, Universidad de Antioquia, Medellín, Colombia.	alejandro.guerrero@gna.org.co
Leon	Yudy Milena	Grupo de Neurociencias de Antioquia (GNA), Facultad de Medicina, Universidad de Antioquia, Medellín, Colombia.	yudy.leon@gna.org.co
Ramirez	Laura	Grupo de Neurociencias de Antioquia (GNA), Facultad de Medicina, Universidad de Antioquia, Medellín, Colombia.	laura.ramirez@gna.org.co
Serna	Laura	Grupo de Neurociencias de Antioquia (GNA), Facultad de Medicina, Universidad de Antioquia, Medellín, Colombia.	laura.serna@gna.org.co
Bocanegra	Yamile	Grupo de Neurociencias de Antioquia (GNA), Facultad de Medicina, Universidad de Antioquia, Medellín, Colombia.	yamile.bocanegra@gna.org.co
Levey	Allan I.	Goizueta Alzheimer's Disease Research Center, Department of Neurology, Emory University, Atlanta, GA 30329	alevey@emory.edu
Johnson	Erik C.B	Goizueta Alzheimer's Disease Research Center, Emory University, Atlanta, GA 30329	erik.johnson@emory.edu
Seyfried	Nicholas T.	Goizueta Alzheimer's Disease Research Center, Emory University, Atlanta, GA 30329	nseyfri@emory.edu
Ringman	John	Department of Neurology, Keck School of Medicine of USC, University of Southern California	john.ringman@med.usc.edu
Fagan	Anne M.	Department of Neurology, Washington University in St. Louis	fagana@wustl.edu
Mori	Hiroshi	Osaka Metropolitan University	mori@omu.ac.jp
Masters	Colin	Florey Institute, The University of Melbourne	c.masters@unimelb.edu.au
Noble	James M.	Taub Institute for Research on Alzheimer's Disease and the Aging Brain, G.H. Sergievsky Center, Department of Neurology, Columbia University Irving Medical Center	jn2054@columbia.edu
Sanchez‐Valle	Raquel	Alzheimer's disease and other cognitive disorders group. Neurology Service. Hospital Clínic de Barcelona. FRCB‐IDIBAPS. University of Barcelona, Barcelona (Spain)	RSANCHEZ@clinic.cat
Lopera	Francisco	Grupo de Neurociencias de Antioquia (GNA), Facultad de Medicina, Universidad de Antioquia, Medellín, Colombia.	francisco.lopera@gna.org.co

## References

[1] Mann DMA , Davidson YS , Robinson AC , et al. Patterns and severity of vascular amyloid in Alzheimer's disease associated with duplications and missense mutations in APP gene, Down syndrome and sporadic Alzheimer's disease. Acta Neuropathol. 2018;136:569‐587. doi:10.1007/s00401-018-1866-3 29770843 PMC6132946

[2] Korenberg JR , Pulst S , Neve RL , West R . The Alzheimer amyloid precursor protein maps to human chromosome 21 bands q21.lOS‐q21.05. Genomics. 1989;5:124‐127. doi:10.1016/0888-7543(89)90095-5 2527801

[3] Rovelet‐Lecrux A , Hannequin D , Raux G , et al. APP locus duplication causes autosomal dominant early‐onset Alzheimer disease with cerebral amyloid angiopathy. Nat Genet. 2006;38:24‐26. doi:10.1038/ng1718 16369530

[4] Lott IT , Head E . Dementia in Down syndrome: unique insights for Alzheimer disease research. Nat Rev Neurol. 2019;15:135‐147. doi:10.1038/s41582-018-0132-6 30733618 PMC8061428

[5] Cabrejo L , Guyant‐Marechal L , Laquerriere A , et al. Phenotype associated with APP duplication in five families. Brain. 2006;129:2966‐2976. doi:10.1093/brain/awl237 16959815

[6] Zarea A , Charbonnier C , Rovelet‐Lecrux A , et al. Seizures in dominantly inherited Alzheimer disease. Neurology. 2016;87:912‐919. doi:10.1212/WNL.0000000000003048 27466472

[7] Baksh RA , Pape SE , Chan LiF , Aslam AA , Gulliford MC , Strydom A . Multiple morbidity across the lifespan in people with Down syndrome or intellectual disabilities: a population‐based cohort study using electronic health records. Lancet Public Health. 2023;8:e453‐e462. doi:10.1016/S2468-2667(23)00057-9 37119823

[8] Castro P , Zaman S , Holland A . Alzheimer's disease in people with Down's syndrome: the prospects for and the challenges of developing preventative treatments. J Neurol. 2017;264:804‐813. doi:10.1007/s00415-016-8308-8 27778163 PMC5374178

[9] Wiseman FK , Al‐Janabi T , Hardy J , et al. A genetic cause of Alzheimer disease: mechanistic insights from Down syndrome. Nat Rev Neurosci. 2015;16:564‐574. doi:10.1038/nrn3983 26243569 PMC4678594

[10] Fortea J , Vilaplana E , Carmona‐Iragui M , et al. Clinical and biomarker changes of Alzheimer's disease in adults with Down syndrome: a cross‐sectional study. Lancet. 2020;395:1988‐1997. doi:10.1016/S0140-6736(20)30689-9 32593336 PMC7322523

[11] Iulita MF , Garzón Chavez D , Klitgaard Christensen M , et al. Association of Alzheimer disease with life expectancy in people with down syndrome. JAMA Netw Open. 2022;5:e2212910. doi:10.1001/jamanetworkopen.2022.12910 35604690 PMC9127560

[12] Ryman DC , Acosta‐Baena N , Aisen PS , et al. Symptom onset in autosomal dominant Alzheimer disease. Neurology. 2014;83:253‐260. doi:10.1212/WNL.0000000000000596 24928124 PMC4117367

[13] Fortea J , Quiroz YT , Ryan NS . Lessons from Down syndrome and autosomal dominant Alzheimer's disease. Lancet Neurol. 2023;22:5‐6. doi:10.1016/S1474-4422(22)00437-9 36517171

[14] Grangeon L , Charbonnier C , Zarea A , et al. Phenotype and imaging features associated with APP duplications. Alzheimers Res Ther. 2023;15:93. doi:10.1186/s13195-023-01172-2 37170141 PMC10173644

[15] Farrer LA . Efects of Age, Sex, and Ethnicity on the Association between Aplipoprotein E genotype and alzheimer disease. JAMA. 1997;278:1349‐1356. doi:10.1001/jama.1997.03550160069041 9343467

[16] Pastor P , Roe CM , Villegas A , et al. Apolipoprotein ε4 modifies Alzheimer's disease onset in an E280A PS1 kindred. Ann Neurol. 2003;54:163‐169. doi:10.1002/ana.10636 12891668

[17] Vélez JI , Lopera F , Sepulveda‐Falla D , et al. APOE*E2 allele delays age of onset in PSEN1 E280A Alzheimer's disease. Mol Psychiatry. 2016;21:916‐924. doi:10.1038/mp.2015.177 26619808 PMC5414071

[18] Almkvist O , Johansson C , Laffita‐Mesa J , Thordardottir S , Graff C . APOE epsilon4 influences cognitive decline positively in APP and negatively in PSEN1 mutation carriers with autosomal‐dominant Alzheimer's disease. Eur J Neurol. 2022;29:3580‐3589. doi:10.1111/ene.15536 36039401 PMC9826049

[19] Langella S , Barksdale NG , Vasquez D , et al. Effect of apolipoprotein genotype and educational attainment on cognitive function in autosomal dominant Alzheimer's disease. Nat Commun. 2023;14:5120. doi:10.1038/s41467-023-40775-z 37612284 PMC10447560

[20] Larsen FK , Baksh RA , McGlinchey E , et al. Age of Alzheimer's disease diagnosis in people with Down syndrome and associated factors: results from the Horizon 21 European Down syndrome consortium. Alzheimers Dement. 2024;20:3270‐3280. doi:10.1002/alz.13779 38506627 PMC11095427

[21] Bejanin A , Iulita MF , Vilaplana E , et al. Association of Apolipoprotein E varepsilon4 Allele with clinical and multimodal biomarker changes of Alzheimer disease in adults with Down syndrome. JAMA Neurol. 2021;78:937‐947. doi:10.1001/jamaneurol.2021.1893 34228042 PMC8261691

[22] Gorijala P , Aslam MM , Dang L‐HaT , et al. Alzheimer's polygenic risk scores are associated with cognitive phenotypes in Down syndrome. Alzheimers Dement. 2024;20:1038‐1049. doi:10.1002/alz.13506 37855447 PMC10916941

[23] Bellenguez C , Küçükali F , Jansen IE , et al. New insights into the genetic etiology of Alzheimer's disease and related dementias. Nat Genet. 2022;54:412‐436. doi:10.1038/s41588-022-01024-z 35379992 PMC9005347

[24] Cruchaga C , Del‐Aguila JL , Saef B , et al. Polygenic risk score of sporadic late‐onset Alzheimer's disease reveals a shared architecture with the familial and early‐onset forms. Alzheimers Dement. 2018;14:205‐214. doi:10.1016/j.jalz.2017.08.013 28943286 PMC5803427

[25] Cochran JN , Acosta‐Uribe J , Esposito BT , et al. Genetic associations with age at dementia onset in the PSEN1 E280A Colombian kindred. Alzheimers Dement. 2023;19:3835‐3847. doi:10.1002/alz.13021 36951251 PMC10514237

[26] Tesi N , van der Lee SJ , Hulsman M , et al. Immune response and endocytosis pathways are associated with the resilience against Alzheimer's disease. Transl Psychiatry. 2020;10:332. doi:10.1038/s41398-020-01018-7 32994401 PMC7524800

[27] van der Flier WM , Scheltens P . Amsterdam Dementia Cohort: performing research to optimize Care. J Alzheimers Dis. 2018;62:1091‐1111. 29562540 10.3233/JAD-170850 PMC5870023

[28] Moulder KL , Snider B , Mills SL , et al. Dominantly Inherited Alzheimer Network: facilitating research and clinical trials. Alzheimers Res Ther. 2013;5:48. doi:10.1186/alzrt213 24131566 PMC3978584

[29] Videla L , Benejam B , Barroeta I , et al. The Down Alzheimer Barcelona Neuroimaging Initiative (DABNI) and its contributions to understanding Alzheimer's disease in Down syndrome: a decade of discovery. Alzheimers Dement. 2025;21:e70259. doi:10.1002/alz.70259 40528282 PMC12173842

[30] Pelleri MC , Cicchini E , Petersen MB , et al. Partial trisomy 21 map: ten cases further supporting the highly restricted Down syndrome critical region (HR‐DSCR) on human chromosome 21. Mol Genet Genomic Med. 2019;7:e797. doi:10.1002/mgg3.797 31237416 PMC6687668

[31] Goldberg TE , Huey ED , Devanand DP . Association of APOE e2 genotype with Alzheimer's and non‐Alzheimer's neurodegenerative pathologies. Nat Commun. 2020;11:4727. doi:10.1038/s41467-020-18198-x 32948752 PMC7501268

[32] Chen S , Francioli LC , Goodrich JK , et al. A genomic mutational constraint map using variation in 76,156 human genomes. Nature. 2024;625:92‐100. doi:10.1038/s41586-023-06045-0 38057664 PMC11629659

[33] riskRegression : Risk Regression Models and Prediction Scores for Survival Analysis with Competing Risks (R package version 2025.09.17) [computer program] 2025.

[34] Nacmias B , Latorraca S , Piersanti P , et al. ApoE genotype and familial Alzheimer's disease: a possible influence on age of onset in APP717 Val Ile mutated families. Neurosci Lett. 1995;183:1‐3. doi:10.1016/0304-3940(94)11100-W 7746463

[35] van der Lee SJ , Wolters FJ , Ikram MK , et al. The effect of APOE and other common genetic variants on the onset of Alzheimer's disease and dementia: a community‐based cohort study. Lancet Neurol. 2018;17:434‐444. 29555425 10.1016/S1474-4422(18)30053-X

[36] Sinai A , Mokrysz C , Bernal J , et al. Predictors of age of diagnosis and survival of Alzheimer's disease in down syndrome. J Alzheimers Dis. 2018;61:717‐728. doi:10.3233/JAD-170624 29226868 PMC6004911

[37] Botte A , Potier MC . Focusing on cellular biomarkers: the endo‐lysosomal pathway in Down syndrome. Prog Brain Res. 2020;251:209‐243. 32057308 10.1016/bs.pbr.2019.10.002

[38] Tosh JL , Rhymes ER , Mumford P , et al. Genetic dissection of down syndrome‐associated alterations in APP/amyloid‐beta biology using mouse models. Sci Rep. 2021;11:5736. doi:10.1038/s41598-021-85062-3 33707583 PMC7952899

[39] Sharma A , Chunduri A , Gopu A , Shatrowsky C , Crusio WE , Delprato A . Common genetic signatures of Alzheimer's disease in Down Syndrome. F1000Res. 2020;9:1299. doi:10.12688/f1000research.27096.1 33633844 PMC7871416

