# Supplementary material for: APOE and genetic risk variants influence Alzheimer's disease onset in carriers of an extra copy of APP, with and without Down syndrome
Source: Alzheimers Dement. 2026 Aug 8;22(8):e71738. doi: 10.1002/alz.71738 (PMC13452017; doi:10.1002/alz.71738)
Supplement: Supplementary file 2 [file ALZ-22-e71738-s002.pdf]

SUPPLEMENTARY MATERIAL

SUPPLEMENTARY METHODS **2**

Literature search PubMed 2

Genotyping and imputation procedures 2

Whole-genome sequencing and analysis of Knight-ADRC and DIAN cohort 2

Genetic analysis of the extra *APP* copy 3

Genetic risk score (AD-GRS) calculation 3

SUPPLEMENTARY RESULTS **4**

Literature search of *APP* duplication carriers 4

SUPPLEMENTARY TABLES **5**

Supplementary Table 1. Genetic variants included in the Alzheimer's disease genetic risk score and corresponding weights in each pathway-specific genetic risk score 5

Supplementary Table 2. Interaction terms of *APOE* and AD-GRS associated with age at onset 7

Supplementary Table 3. Meta-analysis of single variant analysis 8

Supplementary Table 4. Predicted median age at onset at different genetic risks stratified by *APP* duplications and Down syndrome 11

Supplementary Table 5. Predicted median age at onset stratified by *APOE* genotype and by *APP* duplications and Down syndrome 12

Supplementary Table 6. Cox-proportional hazard models with hazard ratios for genes located on chromosome 21 with age at onset in *APP* duplication carriers 13

SUPPLEMENTARY FIGURES **14**

Supplementary Figure 1. Cox-proportional hazard model & meta-analysis of age at onset of the stratified Down syndrome cohorts 14

Supplementary Figure 2. Assessment of test statistic calibration and effect-size concordance 15

Supplementary Figure 3. Predicted cumulative incidence curves at different Alzheimer's disease genetic risk scores stratified by *APOE* genotype and by *APP* duplications and Down syndrome ... 16

Supplementary Figure 4. Heat-map of Pearson correlations of each pathway-specific genetic risk score 17

SUPPLEMENTARY REFERENCES **18**

SUPPLEMENTARY METHODS

Literature search PubMed

("DNA Copy Number Variations"[Mesh] OR "Gene Duplication"[Mesh] OR "Mutation"[Mesh] OR "duplication*"[tiab] OR "copy number variant*"[tiab] OR "copy number*"[tiab] OR "sequence variant*"[tiab]) AND ("Alzheimer Disease"[Mesh] OR "EOAD*"[tiab] OR "Alzheimer's dementia*"[tiab] OR "Alzheimer's disease"[tiab] OR "Alzheimer disease*"[tiab] OR "Familial AD*"[tiab] OR "familial autosomal dominant Alzheimer*"[tiab] OR "Cerebral Amyloid Angiopathy"[Mesh] OR "cerebral amyloid angiopathy*"[tiab]) AND ("Amyloid beta-Protein Precursor"[Mesh] OR "APP*"[tiab] OR "amyloid precursor protein*"[tiab]) AND ("Case Reports" [Publication Type] OR "Cohort Studies"[Mesh])

Genotyping and imputation procedures

For all individuals with genetic data, standard genotyping and imputation methods were performed and established quality control (QC) methods were applied.

Individuals with an *APP* duplication (*APP*dup) and individuals from the DABNI cohort were genotyped using Illumina Global Screening Array. We used high-quality genotyping in all individuals (individual call rate >98%, variant call rate >98%). Samples with discordant genetic sex annotation were excluded. In the DABNI cohort, samples with high or low heterozygosity (F coefficient +/- 3 standard deviations (SD) from the mean) were removed. A relatedness analysis was performed and individuals with a first or second degree relative were excluded ($\pi > 0.125$).

Samples collected from Ace Alzheimer Center Barcelona (ACE) and Hospital Universitario de la Princesa in Madrid (HUP) were genotyped as part of the Genome Research at Ace Alzheimer Center (GR@ACE) and Dementia Genetics Spanish Consortium (DEGESCO) genetic initiatives.[1,2] Genotyping was conducted using the Axiom 815K Spanish biobank array (according to manufacturer's instructions - Axiom™ 2.0 Assay Manual Workflow, Thermo Fisher) at the Spanish National Center for Genotyping (CeGEN, Santiago de Compostela, Spain). Genomic data QC included removal of samples with low-quality genotyping (dish QC < 82%, call rate < 98%), excess heterozygosity (F coefficient +3SD from the mean), or high missingness (individual genotyping rate < 97%). Variants with call rate below 95% or deviation from the Hardy–Weinberg equilibrium (P value < 1×10-06) were also excluded. Samples with discordant genetic sex annotation were excluded.

All genotyping data were imputed on all autosomes with the Trans-Omics for Precision Medicine (TOPMed) reference panel[3,4] on the GRCh38 genome build, and variants with low imputation quality ($R^2 < 0.3$) were excluded. For DS individuals, chromosome 21 was excluded from imputation.

Whole-genome sequencing and analysis of Knight-ADRC and DIAN cohort

Whole-genome sequencing was performed on 2,464 extracted DNA samples from Knight-ADRC and DIAN cohorts using the Illumina TruSeq DNA Sample Preparation Kit on Illumina HiSeq2000/Novaseq6000 platforms. The sequencing targeted an average read depth of 30x and was conducted at various sequencing centers including McDonnell Genome Institute (MGI), Uniformed Services University of the Health Sciences (USUHS), Broad Institute, Genentech or Illumina sequencing center. The resulting high-quality reads were aligned with the human reference genome, GRCh38, using Burrows-Wheeler Aligner (v0.7.17).[5] Variant calling was performed according to GATK's (v4.1.1) best practices, resulting in unified VCF files and stringent quality control process (https://software.broadinstitute.org/gatk/best-practices/).[6] We used the GenotypeGVCFs tool in GATK to merge the intermediate genomic VCF (gVCF) files into a unified VCF file containing the Knight-ADRC and DIAN datasets (n=2,464). We used BCFTOOLS to decompose multiallelic variants into bi-allelic variants.[7] We applied GATK variant quality score recalibration (VQSR) to WGS SNVs and insertion/deletions (indels). Only SNVs and indels with a confidence threshold above 99·9% were included in the analysis. Variants located in regions of low complexity were excluded, and variants with a depth (DP) exceeding the average DP plus 5 standard deviations of the WGS dataset were also excluded. We removed non-polymorphic variants and variants that did not fall within the expected allelic balance ratio for heterozygous calls (ABHet=0·25 to 0·75). We then applied several hard filtering criteria to SNVs and indels: (1) quality depth (QD) ≥ 7 for indels and QD ≥ 2 for SNVs, (2) mapping quality (MQ) ≥ 40, (3) FisherStrand (FS) balance ≥ 200 for indels and FS ≥ 60 for SNVs, (4) strand odds ratio (SOR) ≥ 10 for indels and SOR ≥ 3 for SNVs, (5) inbreeding coefficient (IC) ≥ −0·8 for indels, and (6) ReadPosRankSum (RPRS) test to measure the relative position of reference and alternative alleles within the reads: RPRS ≥ −20 for indels and RPRS ≥ −8 for SNVs. These filters resulted in 41,514,968 high quality genetic variants.

Genetic analysis of the extra *APP* copy

APP duplications were detected using different methods. In many French cases, duplications were identified by quantitative multiplex PCR of short fluorescent fragments (QMPSF) which was performed as part of systematic screening in the clinic. In other cases, the high density single nucleotide polymorphism (SNP) arrays were used in which *APP* duplications were detected using the log R ratio (LRR) and B allele frequency (BAF).[8] The LRR and BAF for each SNP were extracted using Illumina's BeadStudio®. Duplications spanning the full coding sequencing of *APP* (GRCh38/hg38 chr21:25880535-26171128 or GRCh37/hg19 chr21:27252861-27543446) were called with the PennCNV software.[9] Individuals in whom the duplication extended across the entire DS critical region were excluded.[10,11]

For Down syndrome individuals, the presence of a complete trisomy 21 was assessed. In the DABNI cohort, this was done using BAF plots in Illumina's Integrated Genomics Viewer (IGV). Samples showing evidence of mosaicism or recombination events were excluded from the analysis. For samples from ACE and HUP, complete or partial trisomy was confirmed by visualizing BAF or LRR profiles in R. Samples with a partial trisomy that did not cover the *APP* gene were excluded.

Genetic risk score (AD-GRS) calculation

Of the 83 SNPs that reached significance in the AD GWAS (Bellenguez and colleagues) after exclusion of the *APOE* locus, six SNPs were removed from all samples.[12] In the French *APP*dup carriers, two SNPS had a low imputation quality (rs141749679: R^2= 0.0005 and rs72824905: R^2 = 0.00088). Two SNPs were not imputed (rs7157106 and rs117618017) due to low SNP density in the region (n=11, cut-off <20 SNPs) in the DS samples. Two SNPs on chromosome 21 (rs2154481 and rs2830489) were excluded because of the presence of the complete trisomy in all individuals with DS. In the seven DIAN samples, one SNP was not imputed and replaced with the average dosage of the variant, imputed to the minor-allele frequency as reported in Genome Aggregation Database (rs62374257).[13]

For the construction of the pathway-specific GRSs, we used the methods previously described.[14] The number of SNPs included in each pGRS is: 60 SNPs for the β-amyloid pGRS, 42 SNPs for the immune response pGRS, 34 SNPs for the endocytosis pGRS, 39 SNPs for the cholesterol/lipid pGRS and 41 SNPs for the angiogenesis pGRS. The *APOE* locus was excluded from all pGRSs.

SUPPLEMENTARY RESULTS

Literature search of *APP* duplication carriers

In the literature, we found 16 articles covering *APP*dup carriers published between 2004 and 2023.[15-30] Articles that included our own probands were excluded.[22-28] One article reported a patient that carried an *APP*dup and a *PSEN1* p.E318G variant.[20] The *PSEN1* polymorphishm was considered not pathogenic and therefore the carrier was included. Of two articles that covered the same *APP*dup families, we used information from both articles.[16,17] One article could not be included due to missing information on *APOE* genotype.[30] One other article was not included because the *APP*dup family had a chromosome 5 copy number gain co-segregated with the *APP*dup which had co-localized to chromosome 18q21.1.[29] Seven articles with in total 24 *APP*dup carriers were included for the statistical analyses.[15-21]

SUPPLEMENTARY TABLES

Supplementary Table 1. Genetic variants included in the Alzheimer's disease genetic risk score and corresponding weights in each pathway-specific genetic risk score

rsID	Chr	Pos	Gene	Effect allele	Other allele	OR	β-amyloid	Vascular	Cholesterol	Immunity	Endocytosis
**rs679515**	1	207577223	*CR1*	T	C	1.14	0.429	0.000	0.143	0.429	0.000
**rs72777026**	2	9558882	*ADAM17*	G	A	1.06	0.387	0.000	0.032	0.581	0.000
**rs17020490**	2	37304796	*PRKD3*	C	T	1.06	0.567	0.100	0.000	0.333	0.000
**rs143080277**	2	105749599	*NCK2*	C	T	1.41	0.438	0.000	0.063	0.250	0.250
**rs6733839**	2	127135234	*BIN1*	T	C	1.19	0.455	0.091	0.227	0.000	0.227
**rs139643391**	2	202878716	*WDR12*	T	TC	0.94	0.200	0.156	0.111	0.111	0.422
**rs10933431**	2	233117202	*INPP5D*	G	C	0.92	0.333	0.000	0.000	0.667	0.000
**rs16824536**	3	155069722	*MME*	A	G	0.92	0.273	0.727	0.000	0.000	0.000
**rs61762319**	3	155084189	*MME*	G	A	1.17	0.273	0.727	0.000	0.000	0.000
**rs3822030**	4	993555	*IDUA*	G	T	0.95	1.000	0.000	0.000	0.000	0.000
**rs6846529**	4	11023507	*CLNK*	C	T	1.05	0.171	0.086	0.029	0.657	0.057
**rs2245466**	4	40197226	*RHOH*	G	C	1.06	0.000	0.000	0.200	0.200	0.600
**rs112403360**	5	14724304	*ANKH*	A	T	1.09	0.000	0.000	0.000	1.000	0.000
**rs62374257**	5	86927378	*COX7C*	C	T	1.08	0.800	0.000	0.200	0.000	0.000
**rs871269**	5	151052827	*TNIP1*	T	C	0.97	0.500	0.000	0.000	0.500	0.000
**rs113706587**	5	180201150	*RASGEF1C*	A	G	1.1	0.700	0.100	0.100	0.000	0.100
**rs6605556**	6	32615322	*HLA-DQA1*	G	A	0.92	0.000	0.000	0.000	1.000	0.000
**rs10947943**	6	41036354	*UNC5CL*	A	G	0.95	0.636	0.055	0.091	0.218	0.000
**rs143332484**	6	41161469	*TREM2*	T	C	1.39	0.317	0.195	0.146	0.244	0.098
**rs75932628**	6	41161514	*TREM2*	T	C	2.48	0.317	0.195	0.146	0.244	0.098
**rs60755019**	6	41181270	*TREML2*	G	A	1.51	NA	NA	NA	NA	NA
**rs7767350**	6	47517390	*CD2AP*	T	C	1.06	0.000	0.000	0.000	0.000	1.000
**rs785129**	6	114291731	*HS3ST5*	T	C	1.04	0.760	0.120	0.040	0.000	0.080
**rs6943429**	7	7817263	*UMAD1*	T	C	1.06	NA	NA	NA	NA	NA
**rs10952097**	7	8204382	*ICA1*	T	C	1.07	0.000	0.067	0.933	0.000	0.000
**rs13237518**	7	12229967	*TMEM106B*	A	C	0.96	0.000	0.000	1.000	0.000	0.000
**rs1160871**	7	28129126	*JAZF1*	G	GTCTT	0.94	1.000	0.000	0.000	0.000	0.000
**rs6966331**	7	37844191	*EPDR1*	T	C	0.95	0.114	0.486	0.143	0.114	0.143
**rs76928645**	7	54873635	*SEC61G*	T	C	0.94	0.000	0.000	1.000	0.000	0.000
**rs7384878**	7	100334426	*SPDYE3*	C	T	0.94	1.000	0.000	0.000	0.000	0.000
**rs11771145**	7	143413669	*EPHA1*	A	G	0.93	0.286	0.000	0.000	0.143	0.571
**rs1065712**	8	11844613	*CTSB*	C	G	1.13	1.000	0.000	0.000	0.000	0.000
**rs73223431**	8	27362470	*PTK2B*	T	C	1.07	0.300	0.075	0.150	0.375	0.100
**rs11787077**	8	27607795	*CLU*	T	C	0.9	0.432	0.136	0.091	0.159	0.182
**rs34173062**	8	144103704	*SHARPIN*	A	G	1.12	0.000	0.000	0.000	1.000	0.000
**rs1800978**	9	104903697	*ABCA1*	G	C	1.07	0.286	0.143	0.143	0.000	0.429
**rs7912495**	10	11676714	*USP6NL*	G	A	1.06	0.236	0.073	0.382	0.109	0.200
**rs7068231**	10	60025170	*ANK3*	T	G	0.96	0.000	0.125	0.875	0.000	0.000
**rs6586028**	10	80494228	*TSPAN14*	C	T	0.92	1.000	0.000	0.000	0.000	0.000
**rs6584063**	10	96266650	*BLNK*	G	A	0.89	0.000	0.000	0.000	1.000	0.000
**rs7908662**	10	122413396	*PLEKHA1*	G	A	0.96	0.189	0.463	0.074	0.232	0.042
**rs10437655**	11	47370397	*SPI1*	A	G	1.06	0.313	0.000	0.000	0.500	0.188
**rs1582763**	11	60254475	*MS4A4A*	A	G	0.91	NA	NA	NA	NA	NA

**rs3851179**	11	86157598	*EED*	T	C	0.89	0.706	0.059	0.059	0.000	0.176
**rs74685827**	11	121482368	*SORL1*	G	T	1.2	0.583	0.042	0.208	0.000	0.167
**rs11218343**	11	121564878	*SORL1*	C	T	0.85	0.583	0.042	0.208	0.000	0.167
**rs6489896**	12	113281983	*TPCN1*	C	T	1.08	0.200	0.200	0.000	0.600	0.000
**rs17125924**	14	52924962	*FERMT2*	G	A	1.09	0.000	0.000	0.000	1.000	0.000
**rs7401792**	14	92464917	*SLC24A4*	G	A	1.04	0.031	0.215	0.692	0.062	0.000
**rs12590654**	14	92472511	*SLC24A4*	A	G	0.93	0.031	0.215	0.692	0.062	0.000
**rs10131280**	14	106665591	*IGH*	A	G	0.93	0.222	0.000	0.000	0.333	0.444
**rs8025980**	15	50701814	*SPPL2A*	G	A	0.96	0.400	0.000	0.000	0.600	0.000
**rs602602**	15	58764824	*MINDY2*	A	T	0.93	0.800	0.067	0.067	0.000	0.067
**rs3848143**	15	64131307	*SNX1*	G	A	1.05	0.667	0.000	0.000	0.000	0.333
**rs12592898**	15	78936857	*CTSH*	A	G	0.95	0.833	0.000	0.000	0.167	0.000
**rs1140239**	16	30010081	*DOC2A*	T	C	0.94	0.615	0.154	0.000	0.231	0.000
**rs889555**	16	31111250	*BCKDK*	T	C	0.96	1.000	0.000	0.000	0.000	0.000
**rs4985556**	16	70660097	*IL34*	A	C	1.07	0.250	0.750	0.000	0.000	0.000
**rs450674**	16	79574511	*MAF*	C	T	0.95	0.491	0.509	0.000	0.000	0.000
**rs12446759**	16	81739398	*PLCG2*	G	A	0.94	0.188	0.063	0.313	0.250	0.188
**rs16941239**	16	86420604	*FOXF1*	A	T	1.1	0.000	0.000	0.000	1.000	0.000
**rs56407236**	16	90103687	*PRDM7*	A	G	1.13	NA	NA	NA	NA	NA
**rs35048651**	17	1728046	*WDR81*	T	TGAG	1.06	0.357	0.214	0.071	0.071	0.286
**rs7225151**	17	5233752	*SCIMP*	A	G	1.08	0.667	0.000	0.333	0.000	0.000
**rs2242595**	17	18156140	*MYO15A*	A	G	0.95	0.639	0.148	0.077	0.071	0.065
**rs5848**	17	44352876	*GRN*	T	C	1.06	0.059	0.294	0.118	0.529	0.000
**rs199515**	17	46779275	*WNT3*	G	C	0.94	0.223	0.520	0.029	0.149	0.080
**rs616338**	17	49219935	*ABI3*	T	C	1.32	0.700	0.100	0.100	0.000	0.100
**rs2526377**	17	58332680	*TSPOAP1*	G	A	0.96	0.000	0.385	0.000	0.538	0.077
**rs4277405**	17	63471557	*ACE*	C	T	0.94	0.625	0.375	0.000	0.000	0.000
**rs12151021**	19	1050875	*ABCA7*	A	G	1.12	0.588	0.118	0.000	0.000	0.294
**rs149080927**	19	1854254	*KLF16*	G	GC	1.06	0.618	0.055	0.218	0.055	0.055
**rs9304690**	19	49950060	*SIGLEC11*	T	C	1.05	0.700	0.100	0.100	0.000	0.100
**rs587709**	19	54267597	*LILRB2*	C	T	1.07	0.800	0.000	0.000	0.200	0.000
**rs1358782**	20	413334	*RBCK1*	A	G	0.94	0.500	0.000	0.000	0.500	0.000
**rs6014724**	20	56423488	*CASS4*	G	A	0.88	0.857	0.000	0.000	0.000	0.143
**rs6742**	20	63743088	*SLC2A4RG*	T	C	0.95	0.300	0.100	0.200	0.400	0.000

This table shows which 77 SNPs are included in our genetic risk score (GRS) calculations. The weights of the pathway-specific GRS (pGRS) of the beta-amyloid, vascular, cholesterol, immunity and endocytosis pathways are shown. When the weight of a SNP is 0, this SNP was not included in that pGRS. The SNPs with NA as weight are not included in any of the pGRSs. *Abbreviations: rsID = reference SNP ID, Chr = chromosome, Pos = position, OR = odds ratio, β = beta.*

Supplementary Table 2. Interaction terms of *APOE* and AD-GRS associated with age at onset

	Cohort	β [SE]	HR [95% CI]	p-value	N
***APOE* ε2 x AD-GRS**	Total cohort	0.08 [0.16]	1.1 [0.79-1.5]	0.62	1028
	APP duplications	0.46 [0.54]	1.6 [0.54-4.6]	0.4	71
	Down syndrome	-0.08 [0.17]	0.92 [0.66-1.3]	0.65	957
**AD-GRS x *APOE* ε4**	Total cohort	-0.12 [0.13]	0.89 [0.69-1.1]	0.36	1028
	APP duplications	0.04 [0.40]	1.0 [0.47-2.3]	0.92	71
	Down syndrome	-0.18 [0.14]	0.84 [0.64-1.1]	0.19	957

Abbreviations: APOE = apolipoprotein, AD-GRS = Alzheimer's disease genetic risk score, β = beta, SE = standard error, HR = hazard ratio, CI = confidence interval, APP = amyloid precursor protein.

Supplementary Table 3. Meta-analysis of single variant analysis

					Meta-analysis					*APP*dup cohort		DABNI cohort		ACE/HUP cohort		GWAS[1]
Chr	Pos	SNP	Allele	Gene	HR [95% CI]	p-value	q-value	I^2	Hetero	Beta	RAF	Beta	RAF	Beta	RAF	Beta
1	207577223	chr1:207577223:T:C_T	T	CR1	1.1 [0.91-1.3]	0.31	0.78	58.66	0.089	0.25	0.23	-0.15	0.19	0.31	0.23	0.13
2	9558882	chr2:9558882:A:G_G	G	ADAM17	0.97 [0.79-1.2]	0.74	0.94	58.96	0.087	-0.54	0.19	0.08	0.14	0.06	0.19	0.06
2	37304796	chr2:37304796:T:C_C	C	PRKD3	1.0 [0.84-1.3]	0.74	0.94	0.00	0.44	0.11	0.11	-0.13	0.17	0.15	0.11	0.06
2	105749599	chr2:105749599:T:C_C	C	NCK2	5.3 [1.2-24]	0.030	0.27	61.49	0.075	5.10	0.01	0.56	0.00	0.85	0.01	0.34
2	127135234	chr2:127135234:C:T_T	T	BIN1	1.1 [0.99-1.3]	0.069	0.41	0.00	1.0	0.13	0.39	0.12	0.38	0.16	0.39	0.17
2	202878716	chr2:202878716:TC:T_T	T	WDR12	1.1 [0.93-1.4]	0.21	0.68	0.00	0.84	0.11	0.14	0.21	0.14	0.07	0.14	-0.06
2	233117202	chr2:233117202:G:C_G	G	INPP5D	0.95 [0.80-1.1]	0.58	0.85	75.56	0.017	-0.65	0.22	0.11	0.25	-0.03	0.22	-0.08
3	155069722	chr3:155069722:G:A_A	A	MME	1.1 [0.75-1.7]	0.58	0.85	0.00	0.92	0.31	0.03	0.07	0.03	0.09	0.03	-0.08
3	155084189	chr3:155084189:A:G_G	G	MME	1.5 [1.0-2.3]	0.031	0.27	0.00	0.39	1.30	0.01	0.48	0.03	0.23	0.01	0.16
4	993555	chr4:993555:G:T_G	G	IDUA	0.99 [0.86-1.1]	0.86	0.94	0.00	0.67	-0.15	0.49	-0.03	0.47	0.05	0.49	-0.05
4	11023507	chr4:11023507:C:T_C	C	CLNK	1.0 [0.87-1.2]	0.95	1.0	0.00	0.79	-0.01	0.30	-0.04	0.31	0.06	0.30	0.05
4	40197226	chr4:40197226:G:C_G	G	RHOH	0.95 [0.81-1.1]	0.48	0.81	5.31	0.35	-0.33	0.32	-0.07	0.40	0.04	0.32	0.06
5	14724304	chr5:14724304:T:A_A	A	ANKH	1.6 [1.2-2.1]	0.0019	0.11	43.29	0.17	0.22	0.07	0.25	0.06	0.81	0.07	0.09
5	86927378	chr5:86927378:T:C_C	C	COX7C	1.1 [0.90-1.2]	0.52	0.82	55.73	0.10	0.37	0.21	-0.12	0.23	0.10	0.21	0.08
5	151052827	chr5:151052827:C:T_T	T	TNIP1	0.93 [0.79-1.1]	0.34	0.80	50.28	0.13	0.10	0.40	-0.26	0.31	0.04	0.40	-0.03
5	180201150	chr5:180201150:G:A_A	A	RASGEF1C	0.87 [0.70-1.1]	0.22	0.68	0.00	0.40	0.07	0.13	-0.31	0.12	-0.04	0.13	0.10
6	32615322	chr6:32615322:A:G_G	G	HLA-DQA1	0.92 [0.74-1.1]	0.48	0.81	0.00	0.86	-0.24	0.09	-0.04	0.13	-0.08	0.09	-0.08
6	41036354	chr6:41036354:G:A_A	A	UNC5CL	1.0 [0.81-1.3]	0.87	0.94	0.00	0.70	0.21	0.13	0.03	0.13	-0.08	0.13	-0.05
6	41161469	chr6:41161469:C:T_T	T	TREM2	0.52 [0.25-1.1]	0.069	0.41	0.00	0.57	-1.40	0.02	-0.38	0.02	-1.01	0.02	0.33
6	41161514	chr6:41161514:C:T_T	T	TREM2	1.0 [0.31-3.4]	1.0	1.0	14.29	0.31	1.54	0.01	1.72	0.00	-0.54	0.01	0.91
6	41181270	chr6:41181270:A:G_G	G	TREML2	1.8 [0.66-4.9]	0.26	0.68	0.00	0.45	1.70	0.01	0.12	0.01	0.44	0.01	0.41
6	47517390	chr6:47517390:C:T_T	T	CD2AP	1.1 [0.95-1.3]	0.18	0.68	54.96	0.11	-0.29	0.25	0.28	0.26	0.13	0.25	0.06
6	114291731	chr6:114291731:T:C_T	T	HS3ST5	0.96 [0.83-1.1]	0.62	0.89	0.00	1.0	-0.04	0.33	-0.02	0.36	-0.06	0.33	0.04
7	7817263	chr7:7817263:T:C_T	T	UMAD1	1.0 [0.91-1.2]	0.49	0.81	56.82	0.10	-0.16	0.44	-0.04	0.39	0.23	0.44	0.06
7	8204382	chr7:8204382:T:C_T	T	ICA1	1.1 [0.90-1.4]	0.29	0.74	0.00	0.54	0.05	0.12	-0.04	0.10	0.24	0.12	0.07
7	12229967	chr7:12229967:C:A_A	A	TMEM106B	1.1 [0.91-1.2]	0.48	0.81	0.00	0.57	-0.13	0.44	0.07	0.42	0.09	0.44	-0.04
7	28129126	chr7:28129126:GTCTT:G_G	G	JAZF1	1.0 [0.86-1.2]	0.82	0.94	0.00	0.57	0.10	0.21	-0.08	0.22	0.11	0.21	-0.06
7	37844191	chr7:37844191:T:C_T	T	EPDR1	0.94 [0.81-1.1]	0.39	0.81	0.00	0.53	-0.22	0.35	0.03	0.36	-0.10	0.35	-0.05
7	54873635	chr7:54873635:C:T_T	T	SEC61G	0.97 [0.78-1.2]	0.76	0.94	38.74	0.20	-0.22	0.11	-0.20	0.13	0.19	0.11	-0.06
7	100334426	chr7:100334426:C:T_C	C	SPDYE3	0.86 [0.73-1.0]	0.060	0.41	0.00	0.42	0.08	0.33	-0.19	0.30	-0.21	0.33	-0.06
7	143413669	chr7:143413669:G:A_A	A	EPHA1	0.94 [0.80-1.1]	0.42	0.81	77.74	0.011	0.41	0.42	-0.27	0.35	-0.05	0.42	-0.07
8	11844613	chr8:11844613:G:C_C	C	CTSB	1.1 [0.75-1.5]	0.77	0.94	0.00	1.0	0.19	0.02	0.00	0.04	0.06	0.02	0.12
8	27362470	chr8:27362470:C:T_T	T	PTK2B	1.0 [0.91-1.2]	0.56	0.85	0.00	0.91	0.09	0.32	0.06	0.39	0.00	0.32	0.07
8	27607795	chr8:27607795:T:C_T	T	CLU	0.84 [0.72-0.98]	0.023	0.26	0.00	0.60	-0.19	0.39	-0.08	0.38	-0.26	0.39	-0.11
8	144103704	chr8:144103704:G:A_A	A	SHARPIN	0.98 [0.73-1.3]	0.88	0.94	0.00	0.89	-0.16	0.08	0.05	0.08	-0.03	0.08	0.11

9	104903697	chr9:104903697:C:G_G	G	ABCA1	1.1 [0.93-1.4]	0.20	0.68	0.00	0.40	0.35	0.12	0.24	0.13	-0.01	0.12	0.07
10	11676714	chr10:11676714:A:G_G	G	USP6NL	0.96 [0.83-1.1]	0.61	0.89	54.63	0.11	0.02	0.44	0.11	0.48	-0.22	0.44	0.06
10	60025170	chr10:60025170:T:G_T	T	ANK3	0.88 [0.76-1.0]	0.11	0.52	0.00	0.64	-0.08	0.32	-0.05	0.41	-0.21	0.32	-0.04
10	80494228	chr10:80494228:C:T_C	C	TSPAN14	0.86 [0.70-1.0]	0.13	0.55	0.00	0.81	-0.20	0.18	-0.09	0.19	-0.23	0.18	-0.08
10	96266650	chr10:96266650:G:A_G	G	BLNK	1.0 [0.76-1.4]	0.80	0.94	22.31	0.28	0.73	0.05	-0.07	0.05	-0.01	0.05	-0.12
10	122413396	chr10:122413396:A:G_G	G	PLEKHA1	0.99 [0.85-1.1]	0.85	0.94	43.41	0.17	-0.37	0.46	0.06	0.47	0.02	0.46	-0.04
11	47370397	chr11:47370397:G:A_A	A	SPI1	1.1 [0.92-1.2]	0.39	0.81	0.00	0.66	-0.12	0.39	0.09	0.40	0.09	0.39	0.06
11	60254475	chr11:60254475:G:A_A	A	MS4A4A	0.89 [0.77-1.0]	0.11	0.52	0.00	0.47	-0.09	0.45	-0.02	0.38	-0.21	0.45	-0.09
11	86157598	chr11:86157598:T:C_T	T	EED	0.85 [0.72-1.00]	0.049	0.38	16.33	0.30	-0.31	0.32	-0.25	0.35	-0.01	0.32	-0.12
11	121482368	chr11:121482368:T:G_G	G	SORL1	1.5 [0.82-2.6]	0.19	0.68	56.01	0.10	-0.38	0.04	1.27	0.01	0.49	0.04	0.18
11	121564878	chr11:121564878:T:C_C	C	SORL1	1.1 [0.71-1.6]	0.74	0.94	62.60	0.069	1.73	0.01	-0.09	0.02	-0.06	0.01	-0.16
12	113281983	chr12:113281983:T:C_C	C	TPCN1	1.0 [0.79-1.3]	0.84	0.94	0.00	0.66	-0.06	0.08	0.19	0.09	-0.07	0.08	0.08
14	52924962	chr14:52924962:A:G_G	G	FERMT2	1.1 [0.85-1.4]	0.44	0.81	0.00	0.79	-0.07	0.10	0.17	0.07	0.09	0.10	0.09
14	92464917	chr14:92464917:G:A_G	G	SLC24A4	1.0 [0.89-1.2]	0.74	0.94	0.00	0.81	-0.05	0.44	0.01	0.42	0.08	0.44	0.04
14	92472511	chr14:92472511:G:A_A	A	SLC24A4	1.0 [0.88-1.2]	0.80	0.94	0.00	0.78	0.15	0.30	0.01	0.32	-0.02	0.30	-0.07
14	106665591	chr14:106665591:G:A_A	A	IGH	0.99 [0.80-1.2]	0.90	0.94	0.00	0.75	-0.09	0.18	-0.09	0.16	0.07	0.18	-0.07
15	50701814	chr15:50701814:A:G_G	G	SPPL2A	0.81 [0.70-0.94]	0.0052	0.12	39.97	0.19	-0.58	0.32	-0.11	0.37	-0.23	0.32	-0.04
15	58764824	chr15:58764824:T:A_A	A	MINDY2	0.92 [0.78-1.1]	0.37	0.81	0.00	0.69	-0.02	0.25	-0.02	0.23	-0.17	0.25	-0.07
15	64131307	chr15:64131307:G:A_G	G	SNX1	1.0 [0.87-1.2]	0.69	0.93	0.00	0.48	0.12	0.30	-0.09	0.20	0.14	0.30	0.05
15	78936857	chr15:78936857:A:G_A	A	CTSH	0.90 [0.72-1.1]	0.34	0.80	0.00	0.39	-0.15	0.18	-0.26	0.12	0.08	0.18	-0.05
16	30010081	chr16:30010081:C:T_T	T	DOC2A	0.91 [0.78-1.1]	0.21	0.68	10.33	0.33	-0.24	0.35	0.05	0.36	-0.16	0.35	-0.06
16	31111250	chr16:31111250:C:T_T	T	BCKDK	0.95 [0.80-1.1]	0.52	0.82	0.00	0.54	-0.18	0.26	0.04	0.33	-0.13	0.26	-0.04
16	70660097	chr16:70660097:C:A_A	A	IL34	1.4 [1.1-1.8]	0.0061	0.12	44.49	0.17	0.18	0.08	0.13	0.10	0.62	0.08	0.07
16	79574511	chr16:79574511:T:C_C	C	MAF	1.0 [0.89-1.2]	0.64	0.90	0.00	0.86	0.07	0.35	-0.02	0.33	0.07	0.35	-0.05
16	81739398	chr16:81739398:G:A_G	G	PLCG2	1.1 [0.92-1.2]	0.44	0.81	16.07	0.30	-0.10	0.44	0.19	0.42	-0.01	0.44	-0.06
16	86420604	chr16:86420604:T:A_A	A	FOXF1	1.3 [0.86-1.8]	0.24	0.68	0.00	0.55	0.76	0.02	0.03	0.04	0.28	0.02	0.10
16	90103687	chr16:90103687:G:A_A	A	PRDM7	1.1 [0.86-1.5]	0.41	0.81	0.00	0.80	-0.09	0.08	0.18	0.07	0.12	0.08	0.12
17	1728046	chr17:1728046:TGAG:T_T	T	WDR81	1.1 [0.93-1.3]	0.23	0.68	0.00	0.76	0.01	0.23	0.19	0.18	0.09	0.23	0.06
17	5233752	chr17:5233752:G:A_A	A	SCIMP	0.91 [0.73-1.1]	0.40	0.81	0.00	0.48	-0.27	0.08	0.03	0.13	-0.24	0.08	0.08
17	18156140	chr17:18156140:G:A_A	A	MYO15A	0.88 [0.71-1.1]	0.25	0.68	0.00	0.67	-0.04	0.11	-0.24	0.08	-0.04	0.11	-0.05
17	44352876	chr17:44352876:C:T_T	T	GRN	1.2 [1.0-1.4]	0.011	0.18	77.38	0.012	0.82	0.26	0.05	0.30	0.20	0.26	0.06
17	46779275	chr17:46779275:G:C_G	G	WNT3	0.99 [0.84-1.2]	0.87	0.94	24.21	0.27	0.32	0.23	-0.12	0.25	0.00	0.23	-0.06
17	49219935	chr17:49219935:T:C_T	T	ABI3	0.96 [0.29-3.2]	0.95	1.0	0.00	0.68	0.14	0.01	0.93	0.00	-0.65	0.01	0.28
17	58332680	chr17:58332680:A:G_G	G	TSPOAP1	0.89 [0.77-1.0]	0.11	0.52	0.00	0.51	-0.24	0.49	-0.03	0.46	-0.17	0.49	-0.04
17	63471557	chr17:63471557:C:T_C	C	ACE	0.97 [0.84-1.1]	0.69	0.93	0.00	0.55	0.16	0.50	-0.04	0.42	-0.08	0.50	-0.06
19	1050875	chr19:1050875:A:G_A	A	ABCA7	1.3 [1.1-1.5]	0.0029	0.11	0.00	0.73	0.18	0.32	0.16	0.34	0.29	0.32	0.11
19	1854254	chr19:1854254:G:GC_G	G	KLF16	0.88 [0.76-1.0]	0.12	0.52	67.28	0.047	-0.14	0.47	0.11	0.46	-0.30	0.47	0.06
19	49950060	chr19:49950060:C:T_T	T	SIGLEC11	1.1 [0.91-1.3]	0.43	0.81	0.00	0.68	0.11	0.26	0.13	0.23	-0.02	0.26	0.05
19	54267597	chr19:54267597:C:T_C	C	LILRB2	0.94 [0.81-1.1]	0.48	0.81	74.98	0.018	0.52	0.28	-0.08	0.36	-0.22	0.28	0.07

20	413334	chr20:413334:A:G_A	A	RBCK1	1.1 [0.96-1.3]	0.14	0.59	70.24	0.035	0.24	0.30	0.30	0.25	-0.14	0.30	-0.06
20	56423488	chr20:56423488:A:G_G	G	CASS4	1.4 [1.1-1.8]	0.014	0.18	65.35	0.056	0.49	0.08	-0.15	0.07	0.56	0.08	-0.13
20	63743088	chr20:63743088:T:C_T	T	SLC2A4RG	1.0 [0.85-1.2]	0.90	0.94	0.00	0.43	-0.27	0.19	0.06	0.24	0.05	0.19	-0.05

Effect estimates are aligned to the AD-GWAS effect allele. [1]The betas in this column correspond to the betas from the Bellenguez (2022) Alzheimer's disease Genome Wide Association study. Abbreviations: APPdup = amyloid precursor protein duplication, DABNI = Down Alzheimer Barcelona Neuroimaging Initiative, ACE = Ace Alzheimer Center Barcelona, HUP = Hospital Universidad de la Princessa, GWAS = genome wide association study, Chr = chromosome, Pos = position, SNP = single nucleotide polymorphism, HR = hazard ratio, CI = confidence interval, FDR = false discovery rate, I^2 *= Cochran's heterogeneity statistics; Hetero = Cochran Q test p-value, RAF = reference allele frequency.*

Supplementary Table 4. Predicted median age at onset at different genetic risks stratified by *APP* duplications and Down syndrome

	Variable	Median AAO [95% CI]
***APP* duplications**	*APOE* ε2	56.2 [53.9-58.2]
	APOE ε3ε3	52.5 [51.3-53.9]
	APOE ε4	51.1 [49.8-52.3]
	Lowest AD-GRS (-2SD)	54.7 [53.2-56.9]
	Low AD-GRS (-1SD)	53.6 [52.2-55.1]
	Average AD-GRS (0SD)	52.5 [51.3-53.9]
	High AD-GRS (+1SD)	51.5 [50.2-53.0]
	Highest AD-GRS (+2SD)	50.6 [49.3-52.0]
	Lowest risk (*APOE* ε2, lowest AD-GRS)	58.8 [56.5-61.1]
	Highest risk (*APOE* ε4, highest AD-GRS)	49.4 [47.7-50.7]
**Down syndrome**	*APOE* ε2	57.3 [55.1-59.4]
	APOE ε3ε3	53.5 [52.6-54.3]
	APOE ε4	51.8 [51.1-52.8]
	Lowest AD-GRS (-2SD)	56.1 [54.5-57.5]
	Low AD-GRS (-1SD)	54.6 [53.6-55.9]
	Average AD-GRS (0SD)	53.5 [52.6-54.3]
	High AD-GRS (+1SD)	52.4 [51.5-53.4]
	Highest AD-GRS (+2SD)	51.4 [50.4-52.5]
	Lowest risk (*APOE* ε2, lowest AD-GRS)	60.1 [57.6-62.4]
	Highest risk (*APOE* ε4, highest AD-GRS)	50.1 [49.2-51.1]

Abbreviations: AAO = age at onset, CI = confidence interval, APP = amyloid precursor protein, APOE = apolipoprotein, AD-GRS = Alzheimer's disease genetic risk score, SD = standard deviations.

Supplementary Table 5. Predicted median age at onset stratified by *APOE* genotype and by *APP* duplications and Down syndrome

	APOE group	AD-GRS score	Median AAO [95% CI]
***APP* duplications**	*APOE* ε2	Lowest AD-GRS (-2SD)	58.8 [56.5-61.1]
		Low AD-GRS (-1SD)	57.4 [55.1-59.8]
		Average AD-GRS (0SD)	56.2 [53.9-58.2]
		High AD-GRS (+1SD)	54.6 [52.7-57.2]
		Highest AD-GRS (+2SD)	53.5 [51.5-56.0]
	APOE ε3ε3	Lowest AD-GRS (-2SD)	54.7 [53.2-56.9]
		Low AD-GRS (-1SD)	53.6 [52.2-55.1]
		Average AD-GRS (0SD)	52.5 [51.3-53.9]
		High AD-GRS (+1SD)	51.5 [50.2-53.0]
		Highest AD-GRS (+2SD)	50.6 [49.3-52.0]
	APOE ε4	Lowest AD-GRS (-2SD)	53.1 [51.5-54.8]
		Low AD-GRS (-1SD)	51.9 [50.7-53.5]
		Average AD-GRS (0SD)	51.1 [49.8-52.3]
		High AD-GRS (+1SD)	50.2 [49.1-51.4]
		Highest AD-GRS (+2SD)	49.4 [47.7-50.7]
**Down syndrome**	*APOE* ε2	Lowest AD-GRS (-2SD)	60.1 [57.6-62.4]
		Low AD-GRS (-1SD)	58.6 [56.6-60.7]
		Average AD-GRS (0SD)	57.3 [55.1-59.4]
		High AD-GRS (+1SD)	56.0 [53.8-58.0]
		Highest AD-GRS (+2SD)	54.5 [52.5-57.0]
	APOE ε3ε3	Lowest AD-GRS (-2SD)	56.1 [54.5-57.5]
		Low AD-GRS (-1SD)	54.6 [53.6-55.9]
		Average AD-GRS (0SD)	53.5 [52.6-54.3]
		High AD-GRS (+1SD)	52.4 [51.5-53.4]
		Highest AD-GRS (+2SD)	51.4 [50.4-52.5]
	APOE ε4	Lowest AD-GRS (-2SD)	54.1 [52.7-55.7]
		Low AD-GRS (-1SD)	53.0 [51.8-54.0]
		Average AD-GRS (0SD)	51.8 [51.1-52.8]
		High AD-GRS (+1SD)	51.0 [50.1-51.7]
		Highest AD-GRS (+2SD)	50.1 [49.2-51.1]

Abbreviations: AAO = age at onset, CI = confidence interval, APP = amyloid precursor protein, APOE = apolipoprotein, AD-GRS = Alzheimer's disease genetic risk score, SD = standard deviations.

Supplementary Table 6. Cox-proportional hazard models with hazard ratios for genes located on chromosome 21 with age at onset in *APP* duplication carriers

Gene	HR [95% CI]	p-value	q-value	N
A26B3	0.70 [0.28–1.7]	0.44	0.66	92
LOC441956	0.70 [0.28–1.7]	0.44	0.66	92
LIPI	0.76 [0.33–1.7]	0.51	0.66	92
RBM11	0.76 [0.33–1.7]	0.51	0.66	92
STCH	0.68 [0.31–1.5]	0.34	0.66	92
NRIP1	0.68 [0.31–1.5]	0.34	0.66	92
USP25	0.83 [0.41–1.7]	0.60	0.69	92
BTG3	0.78 [0.42–1.4]	0.43	0.66	92
PRSS7	0.78 [0.42–1.4]	0.43	0.66	92
NCAM2	0.83 [0.48–1.4]	0.48	0.66	92
TUBAP1	0.96 [0.58–1.6]	0.87	0.91	92
FLJ42200	1.0 [0.62–1.6]	1.0	1.0	92
C21orf42	0.88 [0.57–1.3]	0.55	0.66	92
MRPL39	1.0 [0.67–1.6]	0.87	0.91	92
JAM2	1.2 [0.71–2.2]	0.44	0.66	92
ATP5J	1.4 [0.77–2.5]	0.27	0.66	92
GABPA	1.6 [0.85–3.1]	0.14	0.66	92
CYYR1	1.3 [0.84–2.0]	0.24	0.66	92
ADAMTS1	1.3 [0.87–2.0]	0.18	0.66	92
ADMATS5	1.2 [0.78–1.8]	0.43	0.66	92
EIF4A1P	1.3 [0.80–2.1]	0.30	0.66	92
USP16	1.8 [0.89–3.6]	0.10	0.66	92
GRIK1	1.7 [0.80–3.8]	0.16	0.66	92

Abbreviations: APP = amyloid precursor protein, HR = hazard ratio, CI = confidence interval, N = number of individuals in analysis.

SUPPLEMENTARY FIGURES

Supplementary Figure 1. Cox-proportional hazard model & meta-analysis of age at onset of the stratified Down syndrome cohorts

	Cohort	N	HR [95% CI]	p-value
**APOE ε2**	DABNI	374	0.86 [0.53-1.4]	0.56
	ACE/HUP	583	0.35 [0.18-0.67]	0.0015
**APOE ε4**	DABNI	374	1.3 [0.90-1.9]	0.16
	ACE/HUP	583	1.6 [1.1-2.3]	0.010
**AD-GRS**	DABNI	374	1.3 [1.1-1.5]	0.0080
	ACE/HUP	583	1.3 [1.1-1.5]	0.0012

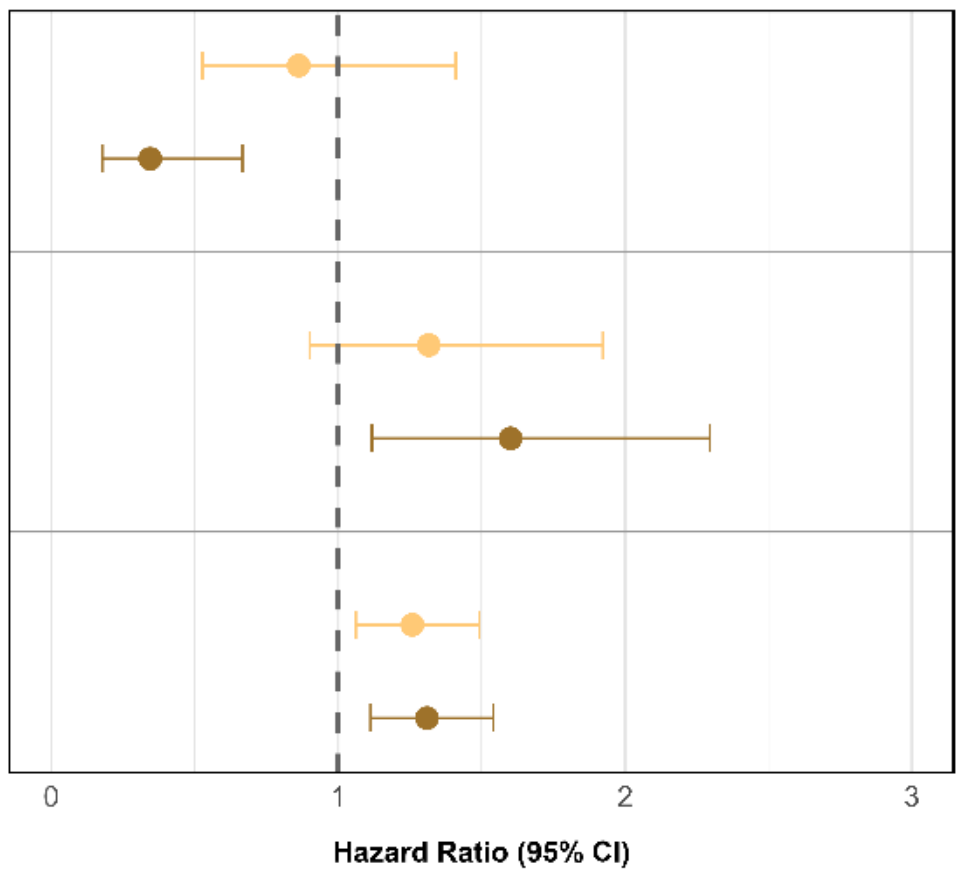

The genetic risk score for Alzheimer's disease (AD-GRS) included 77 SNPs associated with Alzheimer's disease in which the *APOE* genotype was not included. *Abbreviations: N = number of individuals in analysis, HR = hazard ratio, CI = confidence interval, AD-GRS = Alzheimer's disease genetic risk score, APOE = apolipoprotein E, DABNI = Down Alzheimer Barcelona Neuroimaging Initiative, ACE = Ace Alzheimer Center Barcelona, HUP = Hospital Universidad de la Princessa.*

Supplementary Figure 2. Assessment of test statistic calibration and effect-size concordance

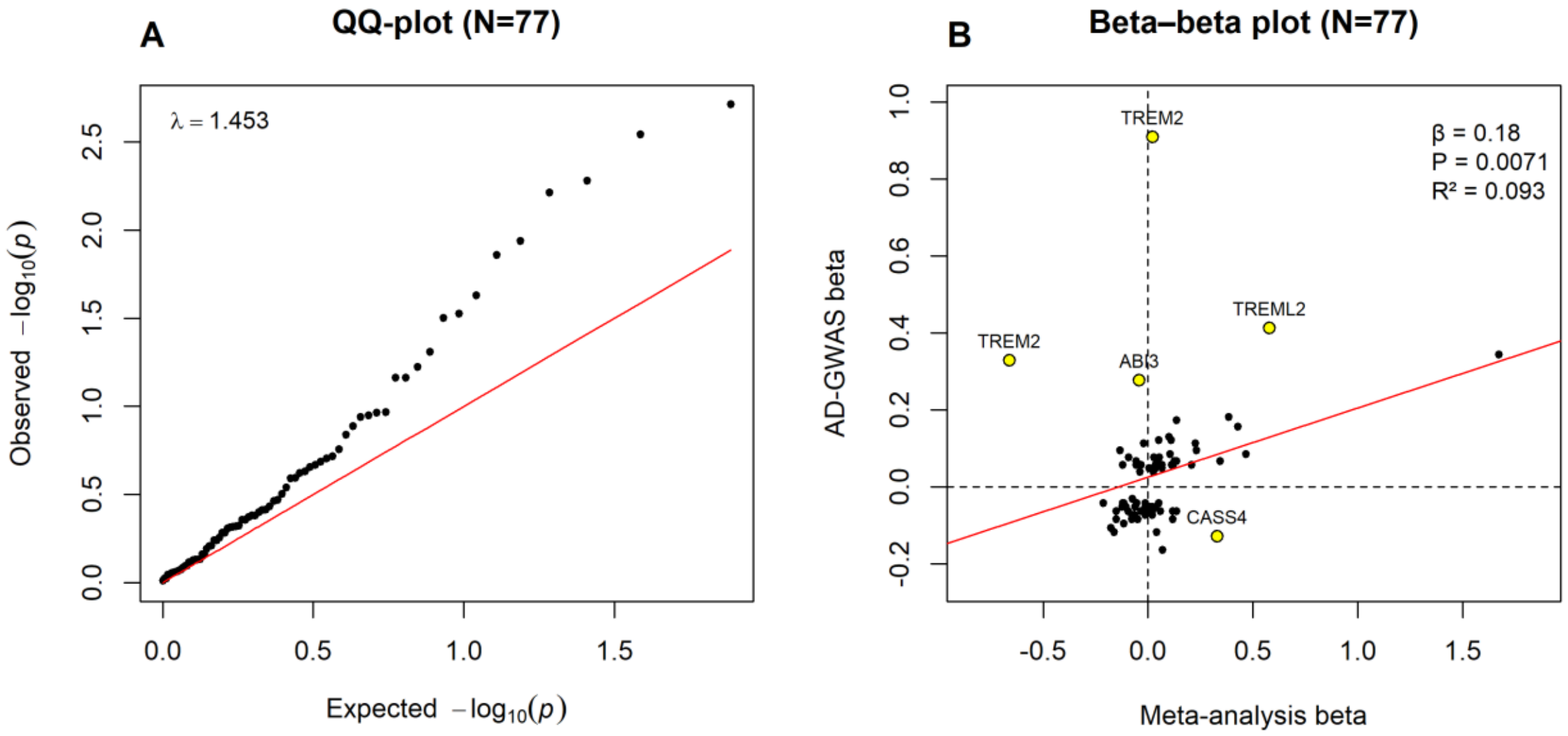

Figure (A) shows the quantile-quantile (QQ) plot of observed versus expected -log10(P) values for the meta-analysis single variant analysis results; genomic inflation factor lambda (λ) is shown. Figure (B) shows the beta-beta plot comparing meta-analysis effect sizes (x-axis) with the Alzheimer's disease genome wide association study (AD-GWAS) effect sizes (y-axis; Bellenguez et al.); the red line indicates the least-squares regression fit.

Supplementary Figure 3. Predicted cumulative incidence curves at different Alzheimer's disease genetic risk scores stratified by *APOE* genotype and by *APP* duplications and Down syndrome

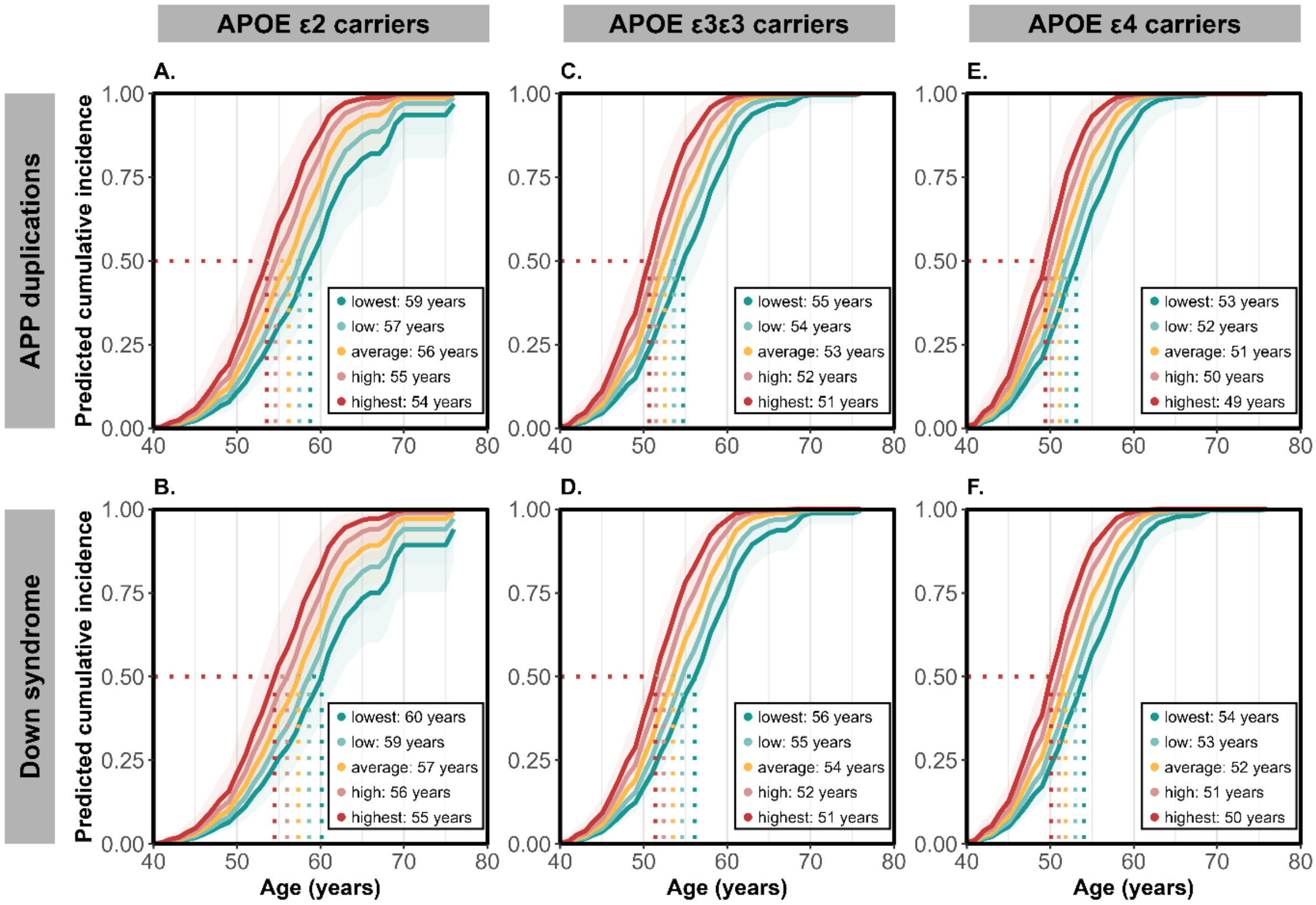

These figures show the predicted cumulative incidence curves stratified by *APP* duplications and Down syndrome and by *APOE* genotype. In Figure (A) and (D), the predicted curves for the different Alzheimer's disease genetic risk scores (AD-GRS) are shown for the *APOE* ε2 carriers (lowest = -2 standard deviations (SD) from the mean AD-GRS, low = -1 SD, average = mean AD-GRS, high = +1 SD, highest = +2 SD). Figure (B) and (E) show the predicted curves for the *APOE* ε3ε3 carriers. In Figure (C) and (D), the predicted curves for the *APOE* ε4 carriers are shown. *Abbreviations: APP = amyloid precursor protein, APOE = apolipoprotein E.*

Supplementary Figure 4. Heat-map of Pearson correlations of each pathway-specific genetic risk score

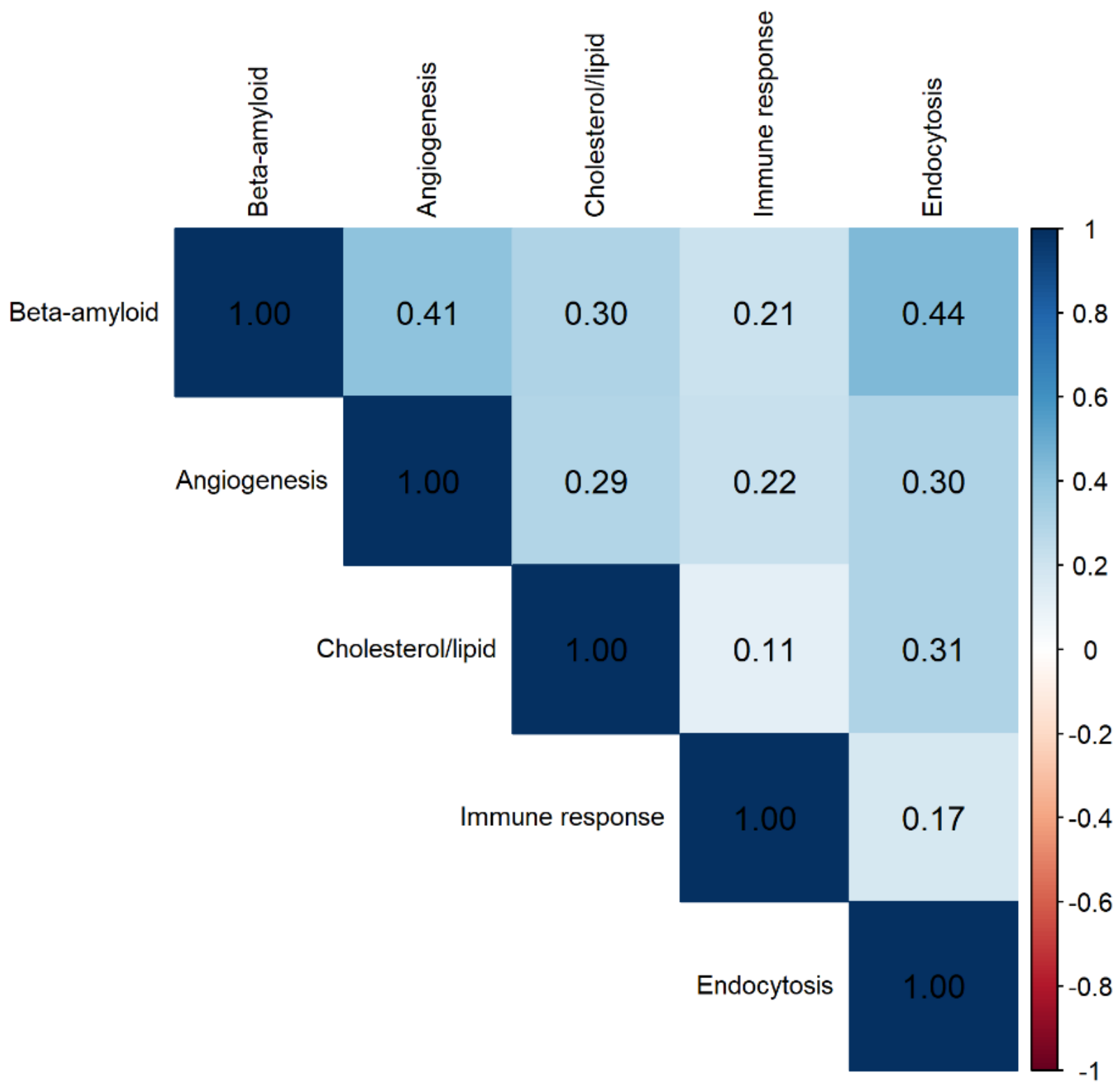

The genetic variants and corresponding weights included in each pathway are shown in *Supplementary Table 1*.

SUPPLEMENTARY REFERENCES

1. Moreno-Grau S, de Rojas I, Hernandez I, et al. Genome-wide association analysis of dementia and its clinical endophenotypes reveal novel loci associated with Alzheimer's disease and three causality networks: The GR@ACE project. *Alzheimers Dement* 2019;15:1333–47.
2. De Rojas I, Moreno-Grau S, Tesi N, et al. Common variants in Alzheimer's disease and risk stratification by polygenic risk scores. *Nat Commun* 2021;12:3417.
3. Das S, Forer L, Schonherr S, et al. Next-generation genotype imputation service and methods. *Nat Genet* 2016;48:1284–87.
4. Taliun D, Harris DN, Kessler MD, et al. Sequencing of 53,831 diverse genomes from the NHLBI TOPMed Program. *Nature* 2021;590:290–99.
5. Li H, Durbin R. Fast and accurate short read alignment with Burrows-Wheeler transform. *Bioinformatics* 2009;25:1754–60.
6. McKenna A, Hanna M, Banks E, et al. The Genome Analysis Toolkit: a MapReduce framework for analyzing next-generation DNA sequencing data. *Genome Res* 2010;20:1297–303.
7. Li H. A statistical framework for SNP calling, mutation discovery, association mapping and population genetical parameter estimation from sequencing data. *Bioinformatics* 2011;27:2987–93.
8. Wang K, Li M, Hadley D, et al. PennCNV: an integrated hidden Markov model designed for high-resolution copy number variation detection in whole-genome SNP genotyping data. *Genome Res* 2007;17:1665–74.
9. Fang L, Wang K. Identification of Copy Number Variants from SNP Arrays Using PennCNV. In: Bickhart DM, ed. Copy Number Variants: Methods and Protocols. New York: *Springer* 2018: 1–28.
10. Pelleri MC, Cicchini E, Locatelli C, et al. Systematic reanalysis of partial trisomy 21 cases with or without Down syndrome suggests a small region on 21q22.13 as critical to the phenotype. *Hum Mol Genet* 2016;25:2525–38.
11. Pelleri MC, Cicchini E, Petersen MB, et al. Partial trisomy 21 map: Ten cases further supporting the highly restricted Down syndrome critical region (HR-DSCR) on human chromosome 21. *Mol Genet Genomic Med* 2019;7:e797.
12. Bellenguez C, Kucukali F, Jansen IE, et al. New insights into the genetic etiology of Alzheimer's disease and related dementias. *Nat Genet* 2022;54:412–36.
13. Karczewski KJ, Francioli LC, Tiao G, et al. The mutational constraint spectrum quantified from variation in 141,456 humans. *Nature* 2020;581:434–43.
14. Tesi N, van der Lee SJ, Hulsman M, et al. Immune response and endocytosis pathways are associated with the resilience against Alzheimer's disease. *Transl Psychiatry* 2020;10:332.
15. McNaughton D, Knight W, Guerreiro R, et al. Duplication of amyloid precursor protein (APP), but not prion protein (PRNP) gene is a significant cause of early onset dementia in a large UK series. *Neurobiol Aging* 2012;33:426.e413–21.
16. Rovelet-Lecrux A, Frebourg T, Tuominen H, Majamaa K, Campion D, Remes AM. APP locus duplication in a Finnish family with dementia and intracerebral haemorrhage. *J Neurol Neurosurg Psychiatry* 2007;78:1158–59.
17. Remes AM, Finnila S, Mononen MA, et al. Hereditary dementia with intracerebral hemorrhages and cerebral amyloid angiopathy. *Neurology* 2004;63:234–40.
18. Kasuga K, Shimohata T, Nishimura A, et al. Identification of independent APP locus duplication in Japanese patients with early-onset Alzheimer disease. *J Neurol Neurosurg Psychiatry* 2009;80:1050–52.
19. Hooli BV, Mohapatra G, Mattheisen M, et al. Role of common and rare APP DNA sequence variants in Alzheimer disease. *Neurology* 2012;78:1250–57.
20. Llado A, Grau-Rivera O, Sanchez-Valle R, et al. Large APP locus duplication in a sporadic case of cerebral haemorrhage. *Neurogenetics* 2014;15:145–49.
21. Antonell A, Gelpi E, Sanchez-Valle R, Martinez R, Molinuevo JL, Llado A. Breakpoint sequence analysis of an AbetaPP locus duplication associated with autosomal dominant Alzheimer's disease and severe cerebral amyloid angiopathy. *J Alzheimers Dis* 2012;28:303–08.
22. Sleegers K, Brouwers N, Gijselinck I, et al. APP duplication is sufficient to cause early onset Alzheimer's dementia with cerebral amyloid angiopathy. *Brain* 2006;129:2977–83.
23. Grangeon L, Charbonnier C, Zarea A, et al. Phenotype and imaging features associated with APP duplications. *Alzheimers Res Ther* 2023;15:93.
24. Guyant-Marechal I, Berger E, Laquerrière A, et al. Intrafamilial diversity of phenotype associated with APP duplication. *Neurology* 2008;71:1925–26.
25. Rovelet-Lecrux A, Hannequin D, Raux G, et al. APP locus duplication causes autosomal dominant early-onset Alzheimer disease with cerebral amyloid angiopathy. *Nat Genet* 2006;38:24–26.
26. Cabrejo L, Guyant-Marechal L, Laquerriere A, et al. Phenotype associated with APP duplication in five families. *Brain* 2006;129:2966–76.

27. Wallon D, Rousseau S, Rovelet-Lecrux A, et al. The French series of autosomal dominant early onset Alzheimer's disease cases: mutation spectrum and cerebrospinal fluid biomarkers. *J Alzheimers Dis* 2012;30:847–56.
28. Lanoiselee HM, Nicolas G, Wallon D, et al. APP, PSEN1, and PSEN2 mutations in early-onset Alzheimer disease: A genetic screening study of familial and sporadic cases. *PLoS Med* 2017;14:e1002270.
29. Kalfon L, Paz R, Raveh-Barak H, et al. Familial early-onset alzheimer's caused by novel genetic variant and APP Duplication: a cross-sectional study. *Curr Alzheimer Res* 2022;19:694–07.
30. Thonberg H, Fallstrom M, Bjorkstrom J, Schoumans J, Nennesmo I, Graff C. Mutation screening of patients with Alzheimer disease identifies APP locus duplication in a Swedish patient. *BMC Res Notes* 2011;4:476.
